# A Network of SLC and ABC Transporter and DME Genes Involved in Remote Sensing and Signaling in the Gut-Liver-Kidney Axis

**DOI:** 10.1038/s41598-019-47798-x

**Published:** 2019-08-15

**Authors:** Sara Brin Rosenthal, Kevin T. Bush, Sanjay K. Nigam

**Affiliations:** 1 Departments of Pediatrics, University of California at San Diego, La Jolla, CA 92093-0693 USA; 2 Departments of Medicine, University of California at San Diego, La Jolla, CA 92093-0693 USA; 3 Center for Computational Biology and Bioinformatics, University of California at San Diego, La Jolla, CA 92093-0693 USA

**Keywords:** Gene regulatory networks, Functional clustering

## Abstract

Genes central to drug absorption, distribution, metabolism and elimination (ADME) also regulate numerous endogenous molecules. The Remote Sensing and Signaling Hypothesis argues that an ADME gene-centered network—including SLC and ABC “drug” transporters, “drug” metabolizing enzymes (DMEs), and regulatory genes—is essential for inter-organ communication via metabolites, signaling molecules, antioxidants, gut microbiome products, uremic solutes, and uremic toxins. By cross-tissue co-expression network analysis, the gut, liver, and kidney (GLK) formed highly connected tissue-specific clusters of SLC transporters, ABC transporters, and DMEs. SLC22, SLC25 and SLC35 families were network hubs, having more inter-organ and intra-organ connections than other families. Analysis of the GLK network revealed key physiological pathways (e.g., involving bile acids and uric acid). A search for additional genes interacting with the network identified HNF4α, HNF1α, and PXR. Knockout gene expression data confirmed ~60–70% of predictions of ADME gene regulation by these transcription factors. Using the GLK network and known ADME genes, we built a tentative gut-liver-kidney “remote sensing and signaling network” consisting of SLC and ABC transporters, as well as DMEs and regulatory proteins. Together with protein-protein interactions to prioritize likely functional connections, this network suggests how multi-specificity combines with oligo-specificity and mono-specificity to regulate homeostasis of numerous endogenous small molecules.

## Introduction

Multi-specific “drug” transporters consist of roughly 50–100 solute carrier (SLC) and ATP-binding cassette (ABC) transporters expressed in all epithelial, as well as many non-epithelial, tissues throughout the body^1–3^. This is a subset of the more than 400 SLC and ABC transporters with mono-specificity, oligo-specificity and multi-specificity^4,5^. Together with Phase1 and Phase2 “drug” metabolizing enzymes, these genes are considered critical in the absorption, distribution, metabolism and elimination (ADME) of small molecule drugs and toxins. However, the endogenous physiological role(s) of these ADME genes, expressed in multiple tissues, is only beginning to be explored.

Metabolomics analyses of murine knockouts of individual “drug” transporter genes and metabolic phenotyping of human polymorphisms and associated disease mutations, as well as *in vitro* transport studies, have made it clear that, in addition to drugs and toxins, these transporters regulate the movement and metabolism of signaling molecules (e.g., prostaglandins, cyclic nucleotides, fatty acids, bile acids, hormones); dietary compounds (e.g., vitamins and anti-oxidants such as uric acid, ergothioneine); gut microbiome products, uremic toxins, and, importantly, metabolites that are rate-limiting for many classical biochemical pathways (e.g., TCA cycle intermediates, carnitine)^1^.

As examples, the transporters OAT1 (SLC22A6) and OAT3 (SLC22A8) are perhaps the major multi-specific transporters of small molecule organic anionic drugs handled by the kidney^6,7^. As a result, there has been a great deal of interest by regulatory agencies and industry in evaluating the transport of new and existing renal-excreted drugs (e.g., antibiotics, antivirals, NSAIDs, diuretics) through OAT1 and OAT3, among other SLC and ABC “drug” transporters^8,9^.

However, metabolomics analyses of the OAT1 and OAT3 knockouts indicates that these evolutionarily conserved genes play a central role in regulating systemic and local (renal) physiology by regulating the movement of small molecules with key informational content—such as rate-limiting metabolites (e.g., α-ketoglutarate), signaling molecules (e.g., bile acids), gut microbiome products (e.g., kynurenine), nutrients (e.g., vitamins) and antioxidants (e.g., urate)—between tissues and body fluid compartments (e.g., blood, CSF, urine, bile)^10–13^. That many of the OAT-transported metabolites (e.g., tryptophan derivatives transported across the gut, bile acids, uremic solutes and uremic toxins of chronic kidney disease) are modified by phase I and phase II DMEs (e.g., sulfation, glucuronidation) before being transported by the kidney suggests intimate connections between transporters and DMEs in the gut, liver and kidney. These and other drug transporter genes, as well as closely related genes, may thus play essential roles in small molecule inter-organ communication.

They also appear to play a key role in inter-organismal communication. In a recent analysis of the organic anion transporter, OAT3 (SLC22A8), it was found that this “drug” transporter functions in the movement of endogenous metabolites flowing through the “gut-liver-kidney” axis^10^. This includes the regulation of levels of many gut microbiome-derived metabolites, some of which are associated with multisystem metabolic disease, such as the uremic syndrome of chronic kidney disease^12,14–16^.

The broader theory of how inter-organ and inter-organismal small organic molecule communication (e.g., metabolites, signaling molecules, antioxidants) is regulated by SLC and ABC transporters--differentially expressed in various remotely interacting organs and organisms--has been termed the “Remote Sensing and Signaling Hypothesis”^2^. This theory is increasingly supported, circumstantially, by the types of metabolomics and transport studies described above^1,17–22^.

Thus, there is a need to consider these “ADME genes” (broadly construed) as a whole from the perspective of endogenous physiology; the availability of huge amounts of omics data of many types from multiple tissues under various conditions enables the analyses of networks of interacting SLC and ABC transporters expressed in multiple remotely interacting tissues. Furthermore, it could potentially allow for analysis of their connections to intermediary enzymes, such as the drug metabolizing enzymes (DMEs) as well as mechanisms regulating the expression of these genes in health and disease, such as those involving nuclear receptors. Indeed, many endogenous ligands are shared between subsets of SLC and ABC “drug” transporters, DMEs, and nuclear receptors (e.g., bile acids, fatty acids)^23,24^.

The coexpression of multiple transporters and DMEs in the same or different tissues could reflect unexplored endogenous roles in systemic and local metabolism, inter-organ communication, or uremic toxin handling. But, at present, it is even difficult to generate potential hypotheses as to which multi-specific, oligo-specific^25^ and monospecific SLC and ABC transporters, as well as various DMEs, interact between tissues to achieve homeostasis of hundreds to thousands of endogenous small molecules in tissues, organs, and body fluids (e.g., blood, bile, urine, cerebrospinal fluid). Identifying such potential interactions involving multi-specific, oligo-specific and monospecific transporters and enzymes could be the first step in connecting them to metabolites and signaling molecules involved in inter-organ communication, thereby giving specificity to interorgan communication pathways involving small molecules and ADME genes/proteins.

One approach for identifying such potential interactions is building networks from co-expression analysis^26–28^. Analysis of the co-expression of genes not only provides a basis for the discovery of transcriptional responses that involve coordinated expression of genes, but also as means for identifying those genes which might work in concert in the cell^29^. Such co-expression networks, while not directly implying causal or mechanistic connections, can be used as a further basis for analyzing wet lab data, as we do here. Using this and other analytic approaches, we are able to define a gut-liver-kidney set of DMEs and SLC and ABC transporters, as well as their interaction with other genes, including nuclear receptors that likely form a “remote sensing and signaling network” involved in inter-organ communication via metabolites and signaling molecules between the intestine, liver and kidney. Furthermore, using available tissue-specific knockout transcriptomics data and our own ChIP-seq data, we were able to substantially validate key regulatory pathways implicated in our analyses.

## Results

Although it has been possible, after decades of physiological studies, to connect SLC and ABC transporters, along with certain DMEs, in the same or different tissues for a limited number of metabolites^2,3^, it has been difficult to define such relationships given the discovery of hundreds of transporters and enzymes, many for which the specific function remains unclear, with sometimes unusual patterns of tissue expression. That such relationships exist in the body is indicated by the huge pharmaceutical literature on drug absorption, distribution, metabolism and excretion (ADME)—processes which are dependent on many of these transporters and DMEs. However, except in a few cases—for example, the gut and liver in the entero-hepatic circulation of bile acids or uric acid handling by the kidney and intestine—it has been extremely hard to define endogenous connections for these highly evolutionarily conserved group of genes^1,30–34^. Furthermore, it has not been easy to understand, except in these few paradigmatic cases, how multi-specificity combines with oligo-specificity and mono-specificity to achieve homeostasis of key metabolites, signaling molecules, pro-oxidants, anti-oxidants and other small organic molecules important in health and disease^30,35^.

One strategy to attack this challenging problem is the employment of co-expression analysis and network building^36–38^. Although not indicating mechanism, and while certain individual associations may turn out to be experimentally unverifiable, or related to assumptions of the method, in aggregate this type of analysis seems likely to provide a reasonable portrait of how sets of genes, or gene families (e.g., SLC22, ABCC) might function together in inter-organ communication (e.g., between the intestine, liver and kidney). Furthermore, co-expression analysis enables the implication of other genes that are either involved in enzymatic processes that are parts of the implicated pathways (e.g., Phase 1 or Phase 2 DMEs) or that regulate the genes (e.g., nuclear receptors and other transcriptional regulators). Importantly, it is possible to then test some of these associations using data from wet lab experiments, as we do below.

In co-expression analysis, it is assumed that, if genes are co-expressed across tissues, particularly under multiple conditions, they are more likely to have an endogenous functional relationship than if they were not co-expressed^39^. Co-expressed genes need not be directly connected, but the assumption is that individually they have a similar function in each tissue in which they are expressed. However, clues to function may also be found by looking at the sets of tissues in which each gene pair is expressed.

Two simple cases would be as follows: 1) The expression pattern across multiple tissues is the same (or highly similar) for a set of genes; or 2) The expression pattern across multiple tissues is different for a set of genes. If the expression pattern for a set of genes is the same across multiple tissues, this raises the possibility of functioning in the same or similar pathways in these multiple tissues (a possibility that which would need to be experimentally supported). On the other hand, if the expression pattern is different this would suggest that the genes function in dissimilar pathways in different tissues.

### Co-expression analysis

**Figure 1:**
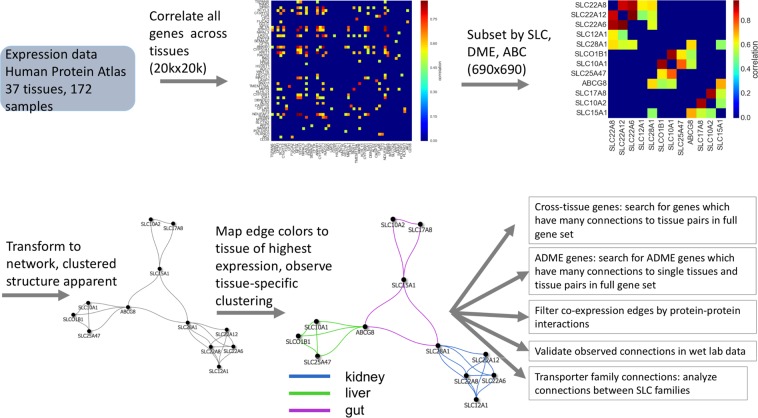
Workflow of the analysis. (1) Expression data from the Human Protein Atlas containing 172 samples across 37 tissue types were collected, and (2) a cross-tissue correlation matrix was created. This correlation matrix was then (3) subset by the transporter-related genes of interest in this study, consisting of SLC, ABC, and DME family genes. This reduced the dimensions of the matrix from 20,000 genes to 690 genes. The correlation matrix was then transformed into network form (4), by treating the genes as nodes, and the correlations as edges, as well as thresholding to include the top 10,000 edges. Thus, if two genes are correlated in the cross-tissue correlation matrix, they will be connected with an edge in the network. Visualization of this network using a spring-embedded layout reveals a significant clustering structure. Additionally, mapping the edge colors (5) to the tissue of highest expression demonstrates that these clusters correspond to specific tissues. From this network we proceed to further downstream analysis (6), by (i) identifying cross-tissue genes: those genes in the full 20,000 gene set which have many connections to pairs of tissues in the reduced network, (ii) validating some of the observed connections in wet lab data, (iii) contextualizing the transporter genes with expert-curated ADME genes, (iv) adding biological relevance by filtering edges by protein-protein interactions, and (v) analyzing the SLC and ABC transporter family connections.

The approach used is schematized in Fig. 1. Co-expression analysis, concentrating on SLC and ABC transporters as well as drug metabolizing enzymes (DMEs), was conducted using the Human Protein Atlas data^40^. While some of these genes are known to participate in metabolite (e.g., glucose, amino acid) transport, these genes (consisting of ~100 families and ~700 genes) include the main gene superfamilies involved in the absorption, distribution, metabolism and elimination (ADME) of small molecule drugs^41^. Many individual family members have been, and continue to be, extensively studied in this regard. From a pharmaceutical perspective, these genes function in a multi-organ system for ADME. Much less is known about the endogenous function of most of these evolutionarily conserved genes and gene families, though it is now becoming clear that certain members function together in a wide range of metabolic pathways and that mutations or SNPs in some of these genes are associated with human metabolic disease^17,41^. It was hypothesized that co-expression analysis might provide insight into the network architecture involving the intra-tissue and inter-organ connections of these genes in a manner highly relevant to their endogenous roles in local and systemic physiology, suggesting new pathways to explore with wet lab studies. Importantly, while some overlap would be expected with genes involved in well-defined ADME pathways in the body, the resulting co-expression network and its topological features would be independent of the pharmaceutical literature and reflect endogenous local and systemic functions. Moreover, based on the Remote Sensing and Signaling Hypothesis, we hypothesized that the genes in the larger network might serve as a scaffold for building a tentative ADME gene-centered “remote sensing and signaling network” that could help guide future experimentation and interpretation of other omics data.

The Human Protein Atlas dataset, which consists of 37 normal human tissues, with transcriptome mRNA data from 172 samples was used to construct a cross-tissue co-expression network. Initially, all 20,000 genes were correlated across all of the various tissues (Fig. 1). This list of all genes was then filtered for Phase I and II drug metabolizing enzymes (DMEs), as well as SLC and ABC transporters (Table S1)—resulting in a total of 690 genes that were used for analysis. While large, this list of transporters and DMEs was not intended to be exhaustive but was meant to capture endogenous interactions and thus some ADME genes prominent in the pharmacology literature may be missing. This set of genes was then transformed into a tissue-specific SLC and ABC transporter and DME network (Fig. 1).

### Tissue of highest average expression for pairs of co-expressed genes mapped to the network

**Figure 2:**
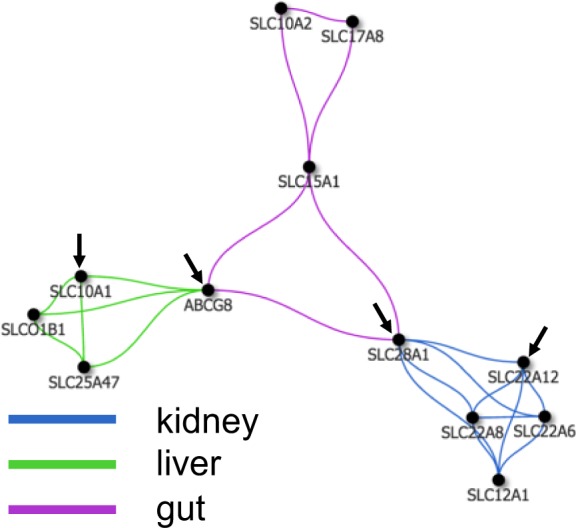
Small portion of the overall network in which the edge colors have been mapped to the tissue of highest expression. The edges in the network are color-coded according to the tissue in which both the source and target nodes have the highest average expression (blue-kidney; green-liver; purple-intestine). The presence of an edge means the two nodes are co-expressed. The color of the edge is based on the tissue in which both source and target nodes are together most highly expressed. This allows one to directly visualize the tissue-specific connections between the various genes and how the genes cluster in the tissues. Arrows indicate individual genes (nodes) that are discussed in the text and how they are connected to other genes.

**Figure 3:**
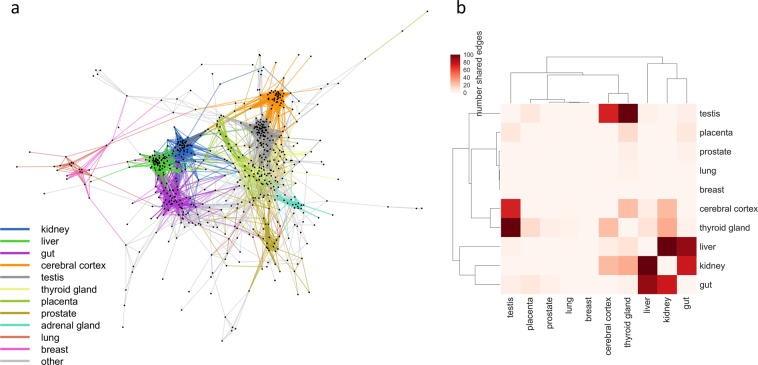
Tissue-specific clusters are apparent in the cross-tissue co-expression network. (**a**) The cross-tissue SLC-ABC-DME correlation network clusters by tissue. Genes positioned using a spring-embedded layout, so that groups highly interconnected genes are positioned near one another. Edges are color-coded by the tissue of highest shared expression by source and target gene. (**b**) Cluster proximity matrix- groups of interconnected tissues. This heatmap displays the number of edges connecting genes with different tissues of highest expression, with rows and columns ordered using hierarchical clustering. The most prominent cluster of tissues is composed of kidney, liver, and gut, suggesting that this group of tissues shares a common set of genes.

In order to visualize the tissue-specific network, it was graphed using a spring-embedded layout with the genes displayed as circular nodes connected by linear edges that were color-coded according to the tissue in which both the source and target nodes have the highest average expression (Figs 2, 3). Displaying the network in this manner highlights those tissues in which pairs of co-expressed genes have the highest expression. For example, the illustration in Fig. 2, may be interpreted as follows: the edges connecting SLC10A1 and ABCG8 indicate that their highest average expression is in the liver, while ABCG8 and SLC28A1 share their highest average expression in the gut, but SLC28A1 and SLC22A12 have their highest average expression in the kidney.

The presumption is that this co-expression reflects related function(s) in the two tissues, an assumption likely to be true in aggregate though not necessarily in each specific instance of co-expression. Pathway enrichment of the tissue-specific genes (Table 1, consisting of 195 genes) shows that the gene assignments, in aggregate, reflect well-established physiological pathways in each tissue: bile acid handling and Phase 1 drug metabolism in the liver; the transport of small organic metabolites and sodium by the kidney; and nutrient transport by the intestine. A later version of the GLK network (used for subsequent network building) consisted of 212 genes (liver--76; kidney--67; gut--67; plus 2 not in any cluster); pathway analysis gave very similar results to those seen in Table 1.

**Table 1:** Pathways enriched in Liver, Kidney, and Gut SLC-ABC-DME genes in one version of the full GLK network.

Tissue	Pathway	q-Value
Liver Genes (N = 76)	Biological oxidations	2.23E-19
	Bile acid and bile salt metabolism	1.39E-15
	Bile secretion	1.03E-12
	Bile acid biosynthesis	4.61E-12
	Primary bile acid biosynthesis	4.61E-12
	Bile acid biosynthesis, neutral pathway	3.07E-11
	Drug metabolism - cytochrome P450	3.72E-11
	Synthesis of bile acids and bile salts	4.55E-11
	SLC-mediated transmembrane transport	6.25E-11
	Synthesis of bile acids and bile salts via 7alpha-hydroxycholesterol	1.35E-10
	Chemical carcinogenesis	2.28E-10
	Phase 1- Functionalization of compounds	2.67E-10
	Transmembrane transport of small molecules	2.08E-09
	Metabolism of xenobiotics by cytochrom P450	2.54E-09
	Phase II conjugation	6.82E-09
	Bile acid biosynthesis, cholestreol = > cholate/chenodeoxycholate	7.04E-09
	MAP00120 Bile acid biosynthesis	9.05E-09
	Synthesis of bile acids and bile salts via 2,4-hydroxycholesterol	2.27E-08
	Recycling of bile acids and salts	6.00E-08
	Metabolic pathways	7.39E-08
Kidney Genes (N = 59)	SLC-mediated transmembrane transport	4.10E-32
	Transmembrane transport of small molecules	1.45E-22
	Transport of glucose and other sugar, bile salts and organic acids, metal ions and amine compounds	6.75E-14
	Transport of inorganic cations/anions and amino acids/oligopeptides	7.45E-14
	Organic cation/anion/zwitterion transport	1.12E-04
	Organic anion transport	1.61E-04
	Amino acid transport across the plasma membrane	1.84E-03
	Type II Na+/Pi cotransporters	1.20E-02
	Na+/Cl-dependent neurotransmitter transporters	1.49E-02
	sodium-coupled sulphate, di- and tri-carboxylate transporters	3.99E-02
Gut Genes (N = 60)	Transmembrane transport of small molecules	1.52E-16
	SLC-mediated transmembrane transport	1.71E-16
	Transport of glucose and other sugar, bile salts and organic acids, metal ions and amine compounds	7.05E-11
	Inositol transporters	1.98E-06
	Bile secretion	2.96E-06
	Transport of inorganic cations/anions and amino acids/oligopeptides	5.19E-05
	Na+-dependent glucose transporters	3.00E-04
	ABC transporters	7.53E-03
	Biological oxidations	8.21E-03
	Trafficking of dietary sterols	1.17E-02

q-value—Bonferroni.

### Gut-Liver-Kidney (GLK) form a highly connected cluster of tissues

Tissue-specific clusters appear in the network (Fig. 3a), which highlight the tissue-specific nature of many genes. These islands of tissue-specific expression include a pronounced set of three highly inter-connected clusters: kidney, liver, and gut. Indeed, by counting the number of between-cluster edges to create a cluster-cluster proximity matrix (Fig. 3b), it was found that kidney, liver, and intestine (which includes small intestine and duodenum) comprise a highly inter-connected set of organs, at least for the co-expression of SLC and ABC drug transporters and DMEs (3B).

**Figure 4:**
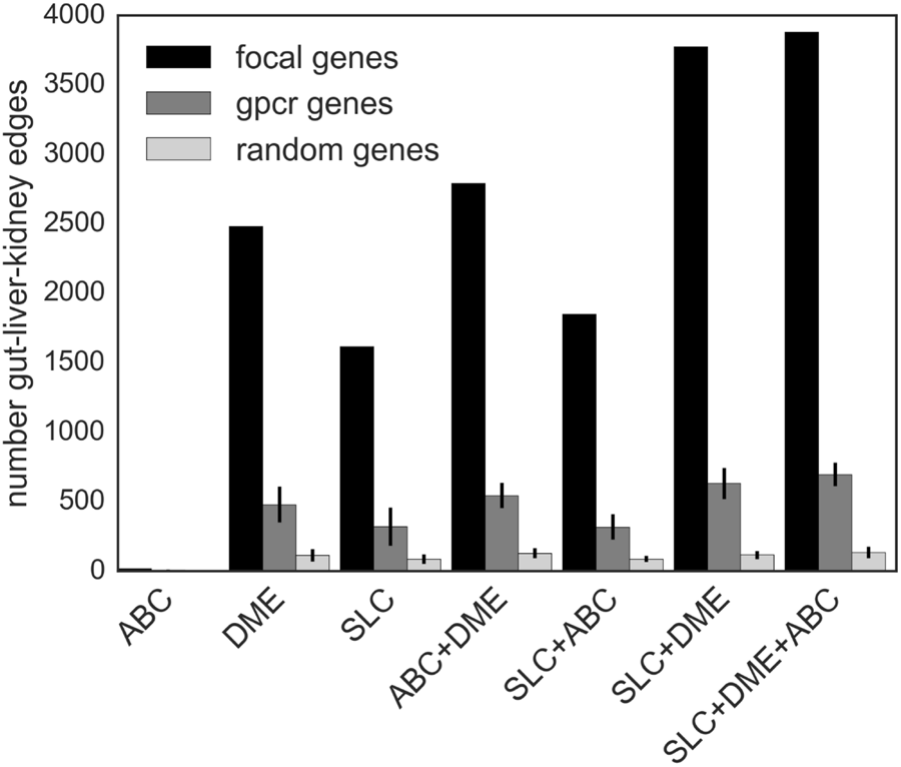
Number of Gut-Liver-Kidney edges are optimized by including SLC, DME, and ABC genes in the subnetwork. No single set of ABC, DME, SLC, or pair of gene sets (i.e., focal genes; black bars) has as many gut-liver-kidney edges as SLC, DME, and ABC together, in the top 10,000 edges. We include randomly selected gene sets of the same size as the focal gene sets (light gray bars), and randomly sampled genes from the set of non-olfactory GPCRs (~3000 in total- dark gray bar) as control sets. We use randomly selected samples that match the size of the focal gene sets. These control gene sets have many fewer gut-liver-kidney edges than the focal gene sets in the top 10000 edges. Random gene distributions were built from 10 samples, and the error bar shows standard deviation.

In this regard, we note that the gut-liver-kidney (GLK) cluster does not appear when creating a network from a randomly selected set of 690 genes (Figs 4, S1), and is highly diminished in a network consisting of 690 GPCRs (Figs 4, S2). Thus we observe that the chosen group of SLC + ABC + DME genes form the strongest gut-liver-kidney cluster of inter-connected genes (Fig. 3a). Additionally, the highest fraction of edges mapping to gut, liver, or kidney are found by including all three gene sets of interest: SLC genes, ABC genes, and DME genes. When considering only single sets (e.g., just SLC genes or just ABC genes), or pairs of sets (e.g., SLC and ABC genes or SLC and DME genes), a smaller fraction of the highest ranking edges map to gut, liver and kidney (Fig. 4). From this one can infer that sets of co-expressed SLC and ABC transporter genes and DMEs are connected across these three tissues, but it remains to be established whether they are involved in similar or different functions in these different tissues.

These three tissues form a distinct cluster, as can be seen in the heatmap, and the associated dendrogram (Fig. 3b). Other clusters include cerebral cortex, testis, thyroid gland, placenta, and prostate; however, some of these unexpected associations might be partly a function of the tissues included in the dataset, and the genes by which the network was restricted. Thus, some of these additional associations may be artificial. On the other hand, all these tissues are known to express important transporters and/or DMEs necessary for tissue and systemic physiology, although they are not nearly as well-studied for this point of view as GLK. The testes is somewhat remarkable in this regard, as many SLC transporters are expressed there, though their actual physiological function is not well understood but may be related to carnitine metabolism necessary for male fertility and protection of developing spermatogonia from drugs and toxins^42–46^. There is  little physiological data on small molecule interactions between these tissues, so they remain worth noting in passing. We also note that the edges between the cerebral cortex and the gut-liver-kidney cluster are relatively sparse compared to the number of edges that cluster to additional tissues, but this interpretation is complicated by the fact that this latter cluster includes many edges falling into the category of “other.”

Previously, co-expression analysis of SLC transporters alone was performed, revealing tissue-specific sets, although certain inter-tissue connections which are so clear in our analysis based on SLCs, ABCs, and DMEs (e.g., gut-liver-kidney) were less apparent in the study based only upon SLCs^26^ (a notion supported by our analysis of SLC genes alone) (Fig. 4). This could be because of well-established physiological data indicating that SLC transporters function in combination with ABC transporters and DMEs in classic metabolic pathways (e.g., bile acids and drug handling).

For example, a recent metabolomics analysis of the organic anion transporter 3 (OAT3, SLC22A8) indicated a role for OAT3 in the movement of metabolites flowing through the “gut-liver-kidney” axis, especially gut microbiome metabolites, bile acids and nutrients that have undergone modification by Phase 2 liver DMEs involved in sulfation and glucuronidation reactions^10^. In this case, the uptake of a metabolite (e.g., dietary component or gut microbiome product) from the gut is mediated by transporters found in intestinal epithelial cells. Once in the blood, the metabolite can be then taken up via transporters into the liver where it is metabolized by “drug” metabolizing enzymes and transported back into the blood via a different set of transporters. This metabolite is ultimately cleared from the blood by basolateral transporters (e.g., OAT1/SLC22A6 and OAT3/SLC22A8) in the proximal tubule of the kidney and secreted via apical transporters into the urine. More limited earlier metabolomics analysis of the Oat1 knockout is also consistent with this view^11,12^.

By the inclusion of DMEs and ABC transporters here, we are able to readily delineate the potential functional connections between the gut, liver and kidney with respect to these particular 690 genes. This co-expression relationship between these three tissues appears to be specific to SLC and ABC transporters and DMEs, as, in a genome-wide analysis, a considerably larger distance was found between liver and kidney^47^. Interestingly, in that study, variation in gene expression was found to be much greater among tissues than among individuals^47^. In this context, it is worth emphasizing again that, when 690 genes were randomly selected from the original set of 20,000, and their co-expression analyzed, the gut-liver-kidney axis is almost absent (Figs 4, S1); this indicates that the co-expressed SLC and ABC transporters and DMEs, but not a randomly selected set of the same size, are intimately associated with the gut-liver-kidney cluster.

### Expanding the network to include genes which are highly connected to gut-liver-kidney SLC, ABC and DME genes

**Figure 5:**
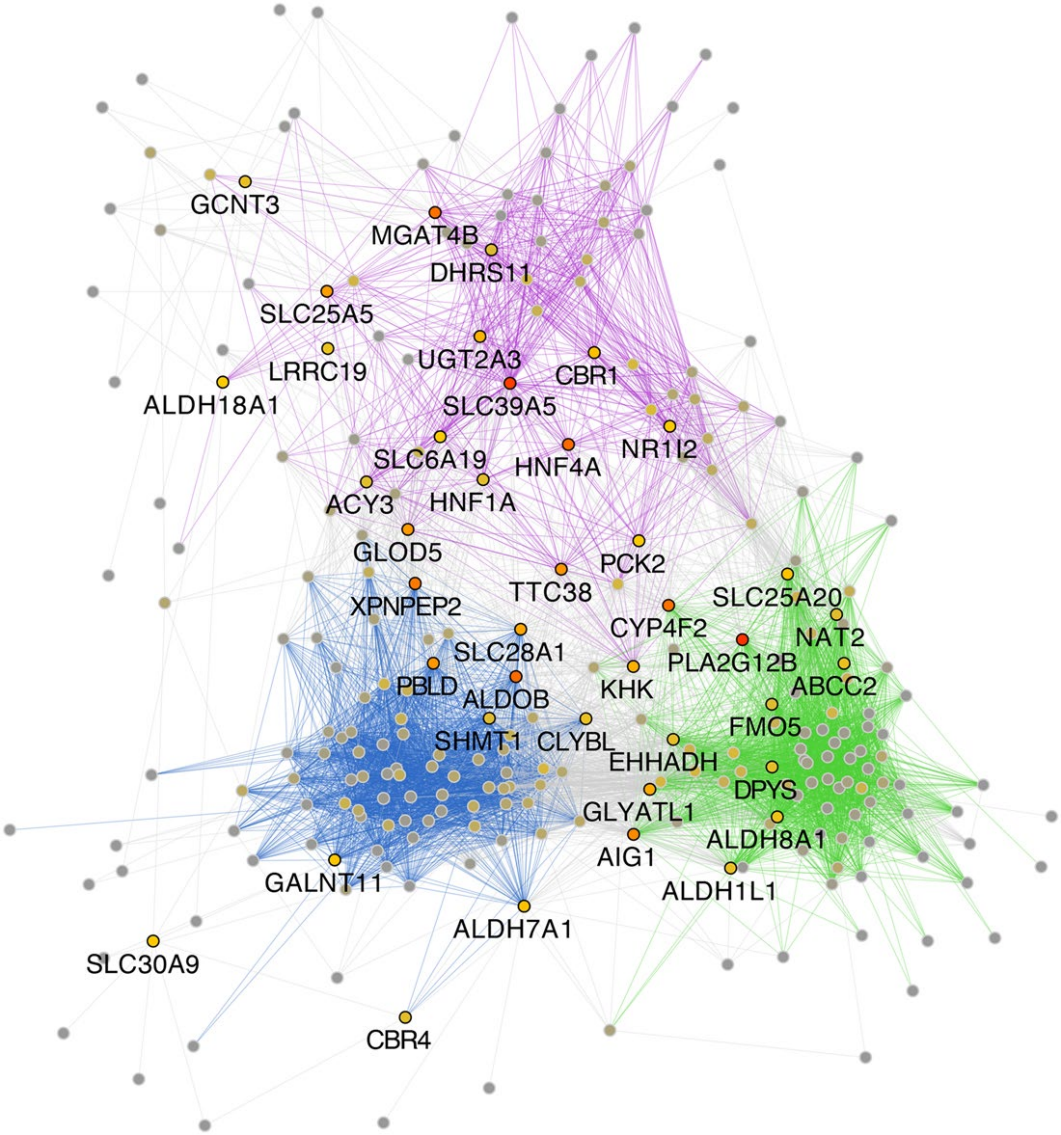
Expanded Gut-Liver-Kidney subnetwork, composed of gut, liver, and kidney expressed genes, and other genes which are highly connected to GLK tissues. This network includes the SLC-ABC-DME genes most highly expressed in one of Gut, Liver, or Kidney, as well as those genes from the entire interactome which are most highly connected to SLC-ABC-DME genes in the Gut-Liver-Kidney subnetwork. Genes are color-coded by their betweenness centrality, while edges remain color-coded by their tissue of highest shared expression.

The genes with the most paired-tissue connections from the full co-expression network (Table S2, Methods) were added to the gut-liver-kidney specific transporter genes to create an expanded subnetwork (Fig. 5). We also calculated the genes with the most single-tissue connections, but did not include them in the expanded network (Table S3, Methods). To bring more biological relevance to the gut-liver-kidney network, edges from protein-protein interaction networks^48^ were incorporated into the gut-liver-kidney network. Specifically, the co-expression edges were filtered by requiring that they also be present in a robust interactome, based on protein-protein interactions. Thus, we extend our tissue-specific framework to data which are functionally relevant—in that the edges in the protein-protein interaction-filtered gut-liver-kidney network likely represent authentic physical interactions. The filtered network retains the tissue-specific clustering which was evident in the co-expression network (Fig. S3). The betweenness centrality for this resulting subnetwork was calculated (Table 2). The betweenness centrality is used as a proxy for prioritizing genes central to multiple tissues, as genes with high betweenness centrality in this subnetwork are the ones which sit at the intersection between tissue clusters (Figs 5, S3, node color), and are thus highly connected to genes from multiple tissues. Prominent among the additional genes in the expanded network were nuclear receptors and other transcriptional factors. Since these transcriptional regulators were deemed likely candidates to be involved in the expression of genes in the SLC, ABC and DME network, relevant available wet lab data (including our own) was interrogated.

**Table 2:** Betweenness Centrality Gut-Liver-Kidney Network.

Gene	Betweenness Centrality
*HNF4A	0.087
ALDOB	0.077
EHHADH	0.049
ABCC2	0.046
SLC2A2	0.042
CYP4F2	0.031
SLC7A9	0.029
XPNPEP2	0.026
AOX1	0.026
ALDH1L1	0.023
*NR1I2	0.023
SLC39A5	0.023
SLC5A1	0.022
CYP2C18	0.021
ACSM5	0.021
AGXT2	0.020
HAO2	0.018
ACSL5	0.018
GLYAT	0.017
SLC22A18	0.017
PCK2	0.017
MGAT4B	0.017
CYP2E1	0.015
CYP27A1	0.015
ECI2	0.015
ALDH8A1	0.015
SLC25A20	0.014
SLC34A1	0.014
SLC3A1	0.014
KHK	0.014
DMGDH	0.013
DPYS	0.013
ASS1	0.012
MTTP	0.012
*HNF1A	0.012

*Transcription factor.

### Wet lab validation of transcription factors with high inter-tissue connections

Coordinate or co-regulation of transporters and/or DMEs in one or more tissues is likely to be mediated by transcription factors and/or nuclear receptors active in these multiple tissues. The betweenness centrality was particularly high for the nuclear receptor HNF4α, when measured in the expanded GLK subnetwork (Table 2), raising the possibility of coordinate upregulation of SLC, ABC and DME genes that are known targets of this nuclear receptor/transcription factor. We therefore reanalyzed existing tissue-specific knockout and our own ChiP-seq data for evidence of a direct link between the coexpression-based results and experimental evidence with respect to HNF4α^49–51^.

Of 108 SLC/ABC transporter or DME genes associated by co-expression analysis with HNF4α in either liver, kidney, or intestine, 69 were down-regulated in either or both HNF4α^−/−^ liver (52 down-regulated) or gut (colon and/or duodenum, 28 down-regulated) (Tables 3, S4). However, HNF4a appears to be required early in kidney development^50,51^. To our knowledge, a late prenatal or perinatal knockout of HNF4a has not been developed, therefore we utilized our previously published ChIP-seq data from kidney^50,51^. By ChIP-seq of kidney cortex, 51 genes or their regulatory regions were noted to have strong peaks for HNF4α (Tables 3, S4); 16 of these did not have altered expression in the liver or intestine knockouts. Thus, in the case of HNF4α, a nuclear receptor with the highest betweenness centrality (Table 2), 85 out of 108 (78.7%) of the significantly-associated transporter and DME genes implicated in the coexpression analysis were supported by wet lab data (Table 3).

**Table 3:** Wet Lab Data Supporting Key Roles of Transcription Factors with High Betweenness Centrality in Gut, Liver Kidney Network of SLC, ABC and DME Genes.

Transcription Factor	Percent Overlap	Accession Number	Reference (PMID)
^a^HNF4α		GSE3126	16714383
		GSE3124	
		GSE3116	16618389
	78.7%	GSE11759	19898610
		GSE34581	22241473
		GSE50815	24038112
^b^HNF1α	61.4%		19289501
^c^PXR	59.3%	GSE55746	26215100

^a^Microarray data from tissue-specific knockouts (liver, colon, small intestine) and ChIP-Seq analysis of adult kidney cortex.

^b^Data from liver of HNF1α knockout.

^c^Data from PXR knockout or from wildtype mice treated with PXR agonist. PXR is NR1I2 from Table 2.

In the analysis of betweenness centrality of the genes in the gut-liver-kidney subnetwork (Fig. 5; Table 2), two other transcription factors were noted. NR1/2, also known as PXR, and HNF1α (Table 2). Thus, these two transcription factors, as with HNF4α, appear to be central nodes in the gut-liver-kidney network. We therefore examined, as with HNF4α, the most highly associated genes with HNF1α (Tables 3, S5) and PXR (Tables 3, S6), and sought to determine if these genes were altered when either of the transcription factors was deleted or chemically activated, as in the case of activation of PXR by the compound PCN^52–54^. Such was the case. In the HNF1α knockout, DME and transporter expression data was combined from several studies that included liver and intestine, and data for 101 of the associated genes was analyzed. Sixty-two of the 101 genes connected to HNF1 α were found to be decreased (~61 percent) (Table 3), strongly supporting our computational analyses. In the case of PXR, 70 of 118 of the connected genes, or 59% were found to be altered in a single tissue (liver) of either the *PxrKO* or following activation of PXR by PCN (Table 3). For HNF4α we had access to omics data for liver, intestine and kidney, while the data we found for HNF1α and PXR was more limited with respect to the gut-liver-kidney axis. Despite this limitation, the corroboration of the computational results with wet lab data seem roughly comparable and lends support to the co-expression approach taken—and the validity of the gut-liver-kidney DME and transporter network that is described above.

### SLC22, SLC25 and SLC35 transporter families are highly connected in the gut-liver-kidney SLC, ABC and DME network

**Figure 6:**
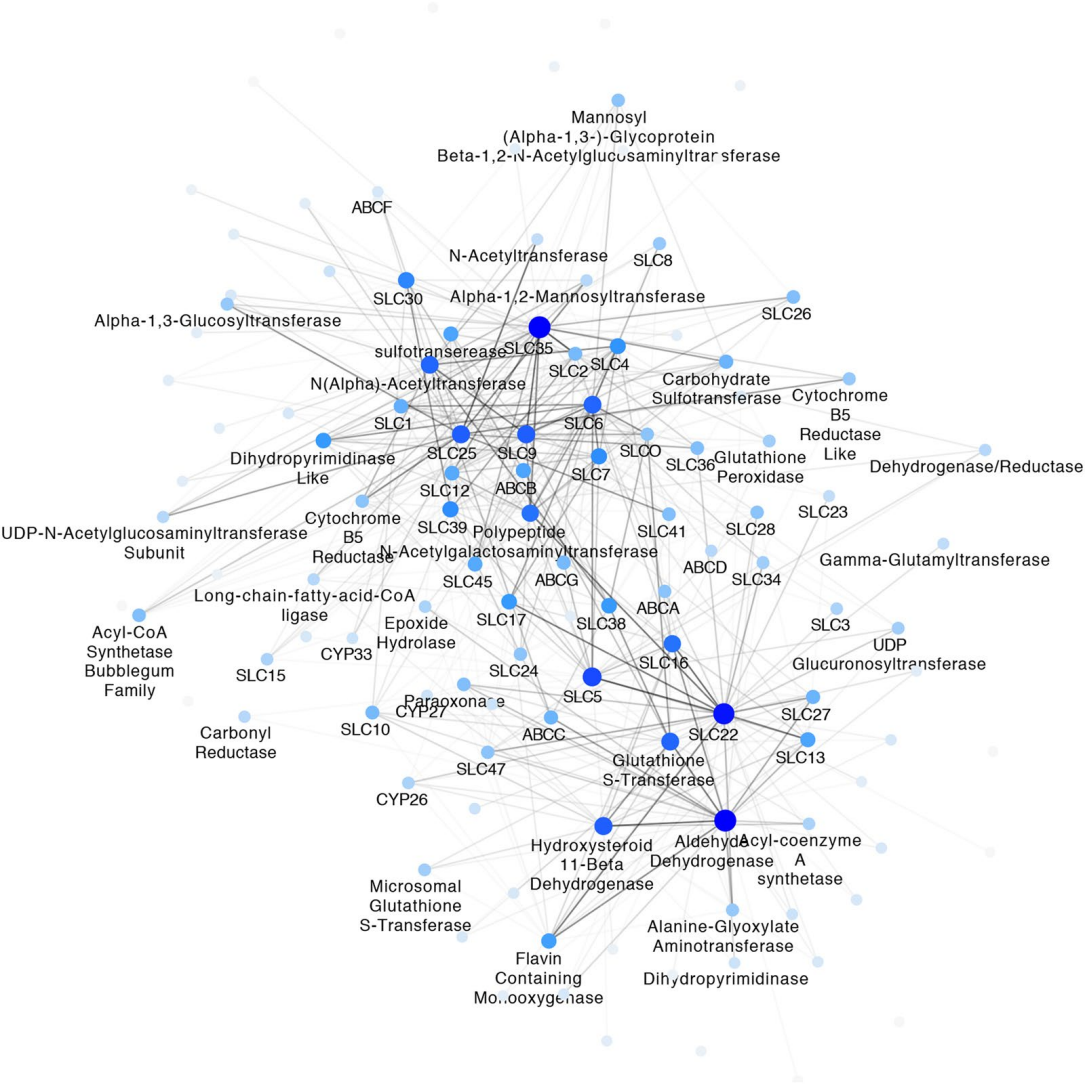
Connections between SLC, DME, and ABC family members. The edge color represents the number of connections between source and target gene family, and edges are only shown if they have a hypergeometric score greater than a threshold of 2. The node color represents the number of significant connections per node (e.g. the node degree), where the dark blue nodes have many connections and the light blue nodes have few connections.

An analysis of the relationships between different transporter families in the co-expression network was performed using all SLC families, ABC families, and by breaking down the DMEs into Phase 1 and 2 enzyme families. By counting the edges between different families or groups a SLC-DME-ABC family gene network was created (Fig. 6). In this network, the edges represent the number of connections which exist between genes in the source node and target node families. To control for the different sizes of gene families, a hypergeometric score between families was calculated to determine if each pair of families has more connecting edges than would be expected by chance. In Fig. 6, we map the number of connections to the edge color, and use the hypergeometric score as an inclusion threshold (where we only include the edge if it has a significant hypergeometric score (p < 0.05)). The node color represents the number of significant connections per node (e.g. the node degree), where the dark blue nodes have many connections, and thus are central and hub-like, and the light blue nodes have few connections, and thus are more peripheral.

In the SLC-DME-ABC family network (Fig. 6), we find that the families group into three distinct clusters. The first cluster is centered around SLC22, Phase 1 and Phase 2 DMEs. The other clusters are centered around SLC35, a family of nucleotide sugar transporters^55,56^, and SLC25, a large family of mitochondrial transporters regulating intermediary metabolism and implicated in diseases affecting a variety of tissues^57,58^. The centrality and high connectivity of these families suggest that they may play an important role in many functions spanning multiple tissues (Table 4).

**Table 4:** Betweenness Centrality SLC and ABC Transporter Families in the full Gut-Liver-Kidney network.

Family Name	Betweenness Centrality
SLC35	0.1899
SLC22	0.1150
SLC25	0.0809
SLC5	0.0569
SLC6	0.0566
SLC30	0.0471
SLC39	0.0461
SLC9	0.0387
SLC16	0.0359
SLC38	0.0291
SLC36	0.0241
ABCC	0.0189
ABCB	0.0187
SLC4	0.0174
SLC26	0.0170
SLC12	0.0170
SLC2	0.0168
SLC10	0.0159
SLC41	0.0148
SLC17	0.0148
SLC7	0.0141
SLC45	0.0123
ABCD	0.0123

The SLC22 family is particularly interesting since it spans organic anion transporters (OAT), organic cation transporters (OCT) and organic carnitine/zwitterion transporters (OCTN)^59–61^. SLC22 transporters are among the best known drug transporters (OAT1, OAT3, OCT1, OCT2), but it is now clear from knockouts (including genome scale metabolic reconstructions of the knockouts) and human SNPs that their endogenous substrates include many metabolites, signaling molecules, gut microbiome products, antioxidants, vitamins and other physiologically important molecules^1,2,60–65^. Several metabolic diseases are associated with mutations or SNPs in SLC22 transporters^2,17,66^. There is a growing amount of evidence that SLC22 transporters such as OAT1 and OAT3 are important in the movement of metabolites through the gut-liver-kidney axis^10,12^.

### Construction of a tentative ADME gene-based remote sensing and signaling network for the gut-liver-kidney axis

We have established that SLC and ABC transporters, together with Phase I and Phase II DMEs as well as key regulatory genes (e.g., nuclear receptors), form a gut-liver-kidney network with highly connected intra-organ nodes, as well as important inter-organ connections. Furthermore, inspection of this network revealed connectedness between multi-specific transporters, including those often called “drug” transporters, with oligo-specific (those which transport a much more limited set of small molecules) and mono-specific transporters. This also appeared to be the case for DMEs.

The Remote Sensing and Signaling Hypothesis holds that inter-organ small molecule communication is mediated by SLC and ABC multi-specific “drug” transporters and multi-specific “drug” metabolizing enzymes in concert with more “oligo-specific” and monospecific transporters and enzymes^14,60,66^; these genes function together to optimize the homeostasis of hundreds if not thousands of endogenous metabolites, signaling molecules, pro-/anti-oxidants, and other small molecules with high informational content in many tissues/organs and body fluid compartments^2,14,60,66^. Careful inspection of the aforementioned gut-liver-kidney network revealed that it contained features of inter-organ communication similar to those which are postulated in the Remote Sensing and Signaling Hypothesis.

As an initial step toward developing such a gut-liver-kidney “remote sensing and signaling network” centered around drug transporters and DMEs, in context with genes that also participate in endogenous physiology involving ADME genes, we analyzed expert-curated lists of ADME genes (www.pharmaadme.org)^67^. These genes are considered to be central to drug handling (“core” list), important to drug handling (“extended: list), or related to drug handling (“related” list). According to the website (www.pharmaadme.org), “An industry initiated effort was launched to develop a consensus, “Core List” of standardized ‘evidence based’ drug metabolizing (ADME) genetic biomarkers that are broadly applicable to many pharmaceutical clinical trials and FDA drug submissions. The effort was driven by a unique multidisciplinary group of representatives from the pharmaceutical industry and an academic center”^67^. The “extended” list—also includes many classical ADME genes such as OAT3 (SLC22A8). Like OAT1 in the “core” list, regulatory agencies include OAT3 in their set of drug transporters that should be considered in the context of pharmacokinetics of new drug entities^59,68^. The “related” list was constructed by considering genes “related to and not directly involved in drug metabolism (www.pharmaadme.org).” This “related” list of genes is not easily categorized, but many genes are clearly involved in endogenous pathways in which the well-accepted ADME genes also participate.

13 “core” and 64 “extended” ADME genes were found within our gut-liver-kidney network of SLCs, ABCs, DMEs and key regulatory molecules (Table S7). Nearly all the genes in the ADME “related “list were not transporters or DMEs, and none were found within the original network. Additionally, ADME genes were over-represented in the set of genes which were most highly connected within and between organ clusters in the GLK network. Within the top 100 genes most connected to the gut-liver-kidney network, transcriptome-wide, we identified 2 additional “core” ADME genes, 21 additional “extended” ADME genes. We also found that 6 genes from the ADME “related” list were highly connected to the transporter genes in the gut-liver-kidney network. Addition of these highly connected genes to the network enhanced the high intra-organ connectivity as well as inter-organ connectivity we observed—leading to a presumptive ADME gene-based “remote sensing and signaling network” for the gut-liver-kidney axis. These new members of the network included many oligo-specific and mono-specific transporters. Focusing on the ADME genes and their first network neighbors enabled us to highlight the non-ADME transporters and enzymes currently known to be involved in a metabolic or signaling pathway that also includes multi-specific transporters and DMEs. In this way, we describe the broader system of interactions between oligo-specific and mono-specific genes involved in bile acid metabolism and uric acid handling, as well as the transport and modification of gut microbe products and uremic toxins, not to mention many other endogenous molecules of physiological importance. Nevertheless, we consider this a tentative network limited in part by the set of ADME genes used to build it, as well as the network strategy employed, and we fully expect it to be revised based on future data and analyses.

### Exemplary genes and pathways in the remote sensing and signaling network

In the remote sensing and signaling network, ALDOB (fructose-1,6-bisphosphate aldolase) an enzyme involved in sugar metabolism and associated with hereditary fructose intolerance, had the highest betweenness centrality (Table 5). Interestingly, this gene was brought into the network via the nearest neighbor expansion of the network. Another gene brought into the network in a similar fashion, with one of the highest betweenness centralities, was EHHADH, an enzyme involved in peroxisome metabolism that is also associated with renal Fanconi symdrome^69^. This suggests that the strategy used for building the network was effective in bringing in non-ADME genes that work together with ADME genes to regulate systemic metabolism. Furthermore, the next most important gene by betweenness centrality was the transcription factor HNF4α, and among transporters, the highest betweenness centrality was for ABCC2 (MRP2)—known to be important in the transport of endogenous organic anions in the liver, kidney and intestine^70^. The next most important transcription factor (after HNF4α) was PXR, and also among the top ten betweenness centralities were two CYP enzymes (CYP4A11 and CYP4F2) (Table 5).

**Table 5:** Top Betweenness Centrality of Genes in the Remote Sensing and Signaling Network (Please also see Supplemental Table S7).

Gene	Betweenness centrality	ADME Genes			First neighbor
		Core	Extended	Related	
ALDOB	0.085				×
HNF4A	0.061		×		
ABCC2	0.053	×			
EHHADH	0.048				×
NR1I2	0.046		×		
CYP4A11	0.042		×		
SLC7A9	0.036				×
CYP4F2	0.035		×		
SLC5A1	0.031				×
HAO2	0.024				×
SLC2A2	0.023				×
AGXT2	0.021				×
ABCG5	0.019				×
ALDH1L1	0.019				×
CES2	0.019		×		
ACSL5	0.019				×
MAT1A	0.018		×		
KHK	0.018				×
DPYS	0.018				×
CYP3A5	0.017	×			
MGAT4B	0.017				×
DAO	0.017				×
SLC28A1	0.017		×		
DMGDH	0.017				×
ACSM5	0.017				×
SOD1	0.016		×		
SLC6A19	0.016				×
NAT2	0.016	×			×
ALDH8A1	0.016		×		
PCK2	0.016				×
XPNPEP2	0.015				×
GLYAT	0.015				×
SLC39A5	0.014				×
SLC25A20	0.014				×
HNF1A	0.014				×

When ranked by degree (number of connecting edges) (Table 6), several CYPs (CYP4A11, CYP2C9, CYP2E1, CYP1A2, CYP8B1, CYP27A1) are included among those genes having degree greater than 30, as well as other Phase I DMEs (FMO3, FMO1, ALDH8A1, ALDH6A1, PON1). Among Phase II DMEs with high degrees were UGT2B4 and NAT2. Among transporters other than ABCC2, a large number of SLC22 family member had high degree (SLC22A1, SLC22A8, SLC22A2, and SLC22A6). Also notable was the presence of SLCO1B1 (OATP1B1).

**Table 6:** High Degree (>30) Genes in Remote Sensing and Signaling Network.

Gene	Degree	ADME Genes			First Neighbor
		Core	Extended	Related	
CYP4A11	58		×		
SLC2A2	57				×
EHHADH	53				×
CYP2C9	53	×			
CYP2E1	52	×			
ADH4	50		×		
ACSM5	49				×
ADH6	49		×		
SLC22A1	47	×			
CYP1A2	46	×			
UGT2B4	45		×		
HSD17B6	45				×
FMO3	45		×		
HAO2	44				×
GLYAT	44				×
ALDH1L1	43				×
AGXT2	42				×
ALDOB	41				×
SLC17A1	41				×
HSD11B1	40		×		
SLC22A8	39		×		
SLC17A3	39				×
ADH1A	39		×		
AKR1D1	39				×
DAO	38				×
DMGDH	38				×
ALDH8A1	38		×		
PON1	38		×		
CYP8B1	38		×		
CAT	37		×		
SLC10A1	37		×		
DPYS	36				×
BAAT	36				×
AGXT	36				×
ABCC2	35	×			
ALDH6A1	35		×		
SLCO1B1	35	×			
SLC22A2	34	×			
SLC12A3	34				×
BHMT	33				×
SLC13A3	33		×		
CYP27A1	33		×		
SLC22A6	33	×			
APOA2	33			×	
SLC3A1	32				×
FMO1	32		×		
NR1I3	32		×		
NAT2	31	×			
SLC34A1	31				×
MAT1A	30		×		
SLC6A13	30				×
SLC38A4	30				×
SERPINA7	30		×		
SLC27A5	30				×

Among SLC family members with high degree brought into the remote sensing and signaling network as first neighbors were SLC17A1 and SLC17A3 (Tables 6, S7), which are known to work together with the ADME genes ABCG2, SLC22A6, SLC22A8, URAT1 (SLC22A12) to regulate uric acid homeostasis^66^. In addition, SLC2A9, which is also important for uric acid homeostasis, was in the network though, like URAT1, it had a degree less than 30 (Tables 6, S7). Similarly, the remote sensing and signaling network captures most transporters involved in various aspects of bile acid handling in different organs, including ABCC2, (MRP2), ABCB11 (BSEP), ABCB4 (MDR2), ABCG8, ABCG5, SLC51A (OSTα), SLC51B (OSTβ), SLCO1B1 (OATP1B1), SLC10A1 (NTCP), SLC10A2 (ASBT), SLC22A8^10,71^. Also present was CYP7A1, which is central to bile acid metabolism^72^. Seven of these were in the ADME gene core or extended list, and six of these were brought into the remote sensing and signaling network as first neighbors (Tables 6, S7). Not only do these examples support the approach we have taken, but they also provide good examples of how multispecific “drug” transporters work together with oligo- and mono-specific transporters to regulate homeostasis of physiologically important metabolites and signaling molecules such as uric acid and bile acids.

Thus, we arrived at, as far as we know, the first, if still somewhat tentative, gut-liver-kidney depiction of a “remote sensing and signaling network” involved in intra-organ and inter-organ communication via SLC and ABC transporters, DMEs, key regulatory genes, and a few other genes that do not fall into any of these categories but are still, based on high connectivity to the network, likely to function in endogenous metabolic and signaling pathways present in the gut, liver and kidney. This “remote sensing and signaling network” contains multi-specific and oligo-specific as well as mono-specific transporters and enzymes. It also contains key regulatory genes which play highly central roles in the network. For example, nuclear receptors like HNF4α, and NR1I2 (PXR) rank in the top 10 genes in the network from the viewpoint of betweenness centrality, indicating that they have an over-representation of edges passing between tissues, and thus may be important for inter-tissue/organ communication. Nevertheless, the remote sensing and signaling network remains in large part a subset of the full gut-liver-kidney network of SLC, ABC and DME genes. Yet, although still centered on so-called ADME genes, it emphasizes how multi-specificity works together with oligo-specificity and mono-specificity to regulate metabolism, signaling and other endogenous pathways within and between organs. It is consistent with what is known about well-described physiological pathways such as bile acid, uric acid and uremic solute handling^2,14,60^; however, it also suggests interactions with genes yet to be studied in these and other physiological contexts.

## Discussion

Many of the SLC and ABC transporters, as well as Phase 1 and Phase 2 DMEs in this set of over 500 genes, are best known for their roles in absorption, distribution, metabolism and elimination (ADME) of small molecule drugs. This is particularly so for the multi-specific transporters (e.g., SLC22, SLCO and certain ABC family members) and various CYPs, UGTs, sulfotransferases (and similar DMEs). For example, among the SLC and ABC “drug” transporters highlighted by regulatory agencies for testing of new drug entities are: SLCO1B1, SLCO1B3, SLC22A2, SLC22A6, SLC22A8, ABCG2 and ABCB1^8,59^. Most academic sources would also include many other SLC and ABC transporter families, such as SLC47 and ABCC. Even many peptide, carnitine and other transporters—for which physiological functions seem well established—are often included in drug transporter lists^2,3^. Indeed, from the pharmaceutical literature and textbooks, one might get the impression that SLC and ABC transporters, along with DMEs, primarily exist—at least at some coordinated systems level—for handling drugs made by pharmaceutical companies via ADME mechanisms. Network reconstructions from knowledge-bases derived from the literature are likely to perpetuate this view, which, based on the analysis we present and the data we cite, may result in a serious misconception.

These genes, and gene families, are highly conserved; orthologs can often be traced to fly, worm, sea urchin and, for some SLC and ABC “drug” transporters, to bacteria^73–76^. Moreover, with the application of metabolomics analyses to knockout mouse body fluids, as well as the advent of genome-based metabolic reconstructions and human SNPs associated with altered metabolite levels, it is now becoming clear that the endogenous function of these transporters and DMEs may very well be to regulate metabolites and signaling molecules in body fluid compartments and small molecule communication between organs (such as the gut, liver and kidney)^2,60,61^. Such a homeostatic system might be as important as classical ones, such as the neuroendocrine system and the growth factor cytokine system^2,30,35,60^. Nevertheless, this set of transporters and DMEs regulating endogenous physiology may have considerable overlap, at the systemic and local level, with the gene network involved in ADME of pharmaceuticals. One might even argue that pharmaceutical drugs are “probes” of this endogenous system, and, simply put, our current understanding of the behavior of the probes is much greater than our understanding of the endogenous system itself^2,60^.

How, then, to arrive at a systems level network representation of the endogenous system which, as with the genes involved in ADME of drugs, is likely to involve multiple organs and pathways for intra-tissue and inter-organ small molecule communication? Building co-expression networks, based on gene expression patterns in multiple tissues might help clarify the physiologically relevant connections of SLC and ABC transporters, together with DMEs, within organs and between organs–in a manner highly relevant to their endogenous physiological function in the regulation of metabolism, signaling and other endogenous processes.

Our analysis of a large, but incomplete list of SLC, ABC and DME genes in the cross-tissue co-expression network revealed strong interconnections between gut, liver and kidney. What is remarkable is that such was not the case for either a random set of genes or another large family of genes known to be of high physiological and pharmacological importance, G protein-coupled receptors (GPCRs), which often bind small molecules transported by SLC and ABC transporters. This indicates a comparatively unique relationship between transporters (SLC and ABC) and DMEs to the gut-liver-kidney axis. A comprehensive search for known regulatory genes which are highly connected to these tissues may shed light on similarities and differences in organ-specific function, and provides a list of potential regulators, for example, transcription factors. In addition, the transporter family-specific network of SLCs, ABCs, and DMEs reveals that several transporter families (eg. SLC35, SLC22, SLC25) are particularly highly connected, having numerous strong connections to other transporter and DME families (Fig. 6).

Many of the gene associations are consistent with physiological and pharmacological data regarding the gut-liver-kidney axis—for example, bile salt handling and the absorption/metabolism/elimination of common organic anion drugs like NSAIDs and β-lactam antibiotics. Importantly, once this network was built, we were able to link additional regulatory genes to the 690 SLC and ABC transporters and DMEs. Among those that had high betweenness centrality in the network including these new genes were transcriptional regulators, including several nuclear receptors. HNF4α was one such nuclear receptor, and analysis of existing knockout data in liver and gut, as well as our own previous ChiP-seq data from kidney, revealed that nearly 70% of the genes significantly associated with HNF4α could be validated with wet lab data. Likewise, ~60% of the predicted associations of the transcription factors HNF1α and PXR (NR1/2)—which also had high betweenness centrality in the new network (SLC, ABC, DME plus new connections, including regulatory genes)—were validated based on published gene expression in knockout tissues or experiments where animals were treated with an activator of a transcription factor.

It seems reasonable to assume that co-expressed genes and proteins are more likely to act together and have an endogenous functional relationship than if they were not co-expressed. In other words, sets of genes which are highly co-expressed, while not directly connected, may play a similar role in each tissue in which they are expressed, and represent a functional module^26^. Thus, while our co-expression analyses remain associations, it is likely that some of the strongest associations, or those with high betweenness centrality in the reconstructed network, are mechanistic links, as in the case of the SLC and ABC transporters as well as DMEs either regulated by HNF4α/HNF1α/PXR (as suggested by the tissue-specific knockout data) or have potential gene regulatory sites to which a nuclear receptor (e.g., HNF4α) is bound (as indicated by our previous kidney ChiP-seq data). These results suggest that our network is likely to reveal other important mechanistic links and can be mined and tested in this way in future studies.

It is worth mentioning in passing that co-expression may “miss” some classical physiological connections. This is in part because, in earlier studies, it was assumed that a transporter or DME had limited expression in one or two tissues and was involved in the handling (e.g., transport, metabolism) of a single chosen “classical substrate.” However, recent expression profiling of many tissues (revealing significant expression in unexpected tissues and cell types) and metabolomics studies of knockout mice may substantially challenge this assumption. For example, ABCC2 (MRP2) is well-established to be involved in bilirubin handling in the liver (in humans and mice), whereas in the kidney it appears to be primarily involved in the apical (luminal) efflux of organic anions taken up by SLC22A6 (OAT1) and SLC22A8 (OAT3)^77^. Another way of looking at this is that the classical physiological studies may have revealed the primary functional relationship or a special case, but there are a large number of other cases which need to be evaluated. Networks such as the one we have built, and validated in a limited way, are an important step in defining these cases; this should reveal new physiologically-relevant biochemical pathways involved in intraorgan, interorgan and interorganismal small molecule remote communication^2,30,35,60,61^.

**Figure 7:**
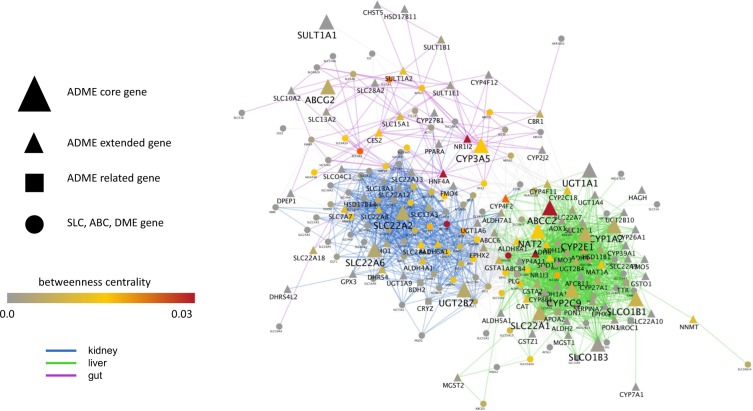
Gut-liver-kidney remote sensing and signaling network focused on ADME genes. This network contains the first neighbors of ADME genes found in the gut-liver-kidney network, with edges filtered by protein-protein interactions (only including edges which are both co-expressed and are found in the protein-protein interaction database). ADME core and extended genes are triangles (large and small respectively). ADME related genes are squares. SLC, ABC, and DME neighbors of ADME genes are circles. The node color encodes the network betweenness centrality. Highly central nodes have more inter-tissue connections. Edge colors are mapped to gut (purple), liver (green), and kidney (blue) specific interactions in the remote sensing and signaling network.

Finally, a major result of our studies is the identification of a “remote sensing and signaling network” involving SLC and ABC transporters, DMEs, key regulatory and other proteins in the gut-liver-kidney axis (Fig. 7). We consider this network to be somewhat tentative and likely to be revised as data from focused mechanistic studies and omics data continues to come in. However, it can serve as a guide to such experimentation as well as the analysis of results. According to the Remote Sensing and Signaling Hypothesis, genes/proteins in such a network are essential for remote inter-organ (and potentially inter-organismal) communication via small organic molecules with high informational content^﻿2﻿,﻿60﻿^. Such small molecules include metabolites (eg. citric acid cycle intermediates, bile acid intermediates), signaling molecules (e.g., short chain fatty acids, cyclic nucleotides, prostaglandins), anti-oxidants (e.g., uric acid, ergothionine), and many other small molecules with key functions in endogenous physiology. With an increasing understanding of metabolic and signaling roles of ADME genes that have heretofore been largely considered from a pharmaceutical perspective, and the widespread employment of omics methods to ADME gene knockouts, as well as GWAS studies, there is a growing appreciation of the roles these genes could play in remote inter-organ and inter-organismal communication in health and disease^2,18,19,21^. Furthermore, what our network begins to reveal is the potential of a limited set of multi-specific, oligo-specific and monospecific transporters and enzymes to regulate the homeostasis of hundreds, if not many thousands, of small molecules in multiple tissues and body fluid compartments. How this actually occurs remains a major question in physiology. Our effort provides an initial representation of such a “remote sensing and signaling network” involving the gut-liver-kidney axis as well as a framework for both guiding and interpreting experiments.

## Materials and Methods

The Human Protein Atlas data^40^, which consists of 37 normal human tissues with transcriptome mRNA data from 172 samples, was used to construct the cross-tissue coexpression network. RNAseq normalized counts were downloaded from the human protein atlas website (version 15). Information on how these data were processed may be found in the original human protein atlas manuscript (https://science.sciencemag.org/content/347/6220/1260419). Pearson’s correlation coefficient was calculated for all pairs of genes, across all tissues, such that the value of the correlation can be interpreted as the tendency of two genes to be expressed in similar sets of tissues. A gene co-expression network was created out of the most strongly co-expressed genes, creating an edge between two genes if they had a correlation coefficient greater than 0.59 (and thus selecting the top 10,000 edges)^26^. The interactions in networks with different correlation thresholds (Table S2, S3) were also examined. The network was then filtered using the genes of interest in this study, including genes in the SLC transporter families, genes in the ABC transporter families, as well as drug metabolizing enzymes (DMEs). After filtering, the resulting subnetwork consisted of 690 genes and 10,000 edges, which is used as the basis for further analysis. For a diagram of the analysis workflow, see Fig. 1.

### Number of gut-liver-kidney edges are optimized by including SLC, DME, and ABC genes in the subnetwork

The SLC-DME-ABC subnetwork is densely connected in the gut, liver and kidney tissues. To evaluate this quantitatively, the density of the edges for selected sets of genes was measured and compared to the full set of SLC genes, DME genes, and ABC genes. New subsets of the full coexpression matrix was created by filtering by the genes of interest, and measuring what fraction of the top 10,000 edges belong to the gut, liver or kidney.

It is clear that no single set of ABC, DME, SLC, or pair of gene sets has as many gut-liver-kidney edges as SLC, DME, and ABC together, in the top 10,000 edges. As a control, randomly selected gene sets of the same size as the focal (SLC, DME and/or ABC) gene sets, along with randomly sampled genes from the set of non-olfactory GPCRs(~3000 in total) were also included. These control gene sets have many fewer gut-liver-kidney edges than the focal gene sets in the top 10,000 edges. Random gene distributions were built from 10 sampling repetitions.

### Single tissue connections

 The number of connections from the three tissues of interest (gut, liver, kidney) in the SLC-DME-ABC genes to the remainder of genes not originally included was calculated, by grouping by tissue, and then summing across the full adjacency matrix, followed by sorting. (Table S3).

### Paired tissue connections

An expanded SLC-DME-ABC subnetwork was created by bringing in the genes most highly intereconnected across tissues (e.g., those genes with the highest number of connections to two separate tissues). The number of connections between all genes and all three pairs of tissues (gut-kidney, gut-liver, and liver-kidney) was first calculated. Next the minimum of each triplet was calculated and the list was sorted by this value, thus recovering genes which have a large number of connections to both tissues, not just one. The top 20 most highly ranking genes from each tissue pair were included in the expanded network (Table S2).

### Filtering by protein-protein interactions

The co-expression network was filtered by protein-protein interactions to prioritize edges with likely biological relevance. We used PCNet, the parsimonious composite network resulting from a recent study comparing the performance of a number of commonly used protein-protein interaction databases^48^. Edges were included if they were found in the drug transporter coexpression network, as well as in the PCNet interactome. The number of edges in the filtered network was thereby reduced to 1959 in contrast to 6036 in the original GLK coexpression network.

### Pathway analysis

Pathway analysis was conducted using the ToppGene analysis suite^78^.

### Wet lab validation

To find evidence of a direct link between co-expression-based analysis and transcriptional regulation, available knockout data for the transcription factors, as well as ChIP-seq data were analyzed as described below.

Briefly, for the analysis of gene expression in wild-type and transcription factor knockout tissues, publically available mRNA expression data were utilized (please see Table 3 for details). The robust multi-array average algorithm and global renormalization^79^ was used to normalize the samples for comparisons between multiple samples generated by different laboratories using different mouse strains. Probes that did not have a present flag in more than half of the samples in at least one of the conditions as determined by the MAS5 algorithm were discarded and a moderated t-test with a Benjamini-Hochberg multiple test correction was used to identify differentially expressed genes (≥100-fold change; p ≤ 0.05) between wildtype and knockout tissues.

The chromatin immunoprecipitation followed by high throughput sequencing (ChIP-seq) for Hnf4α and its analyses has been previously described^50,51^. Briefly, ChIP analyses were performed in duplicate using chromatin prepared from adult Sprague-Dawley rat whole kidneys and kidney cortex. Sequence amplified DNA fragments (200–400 bp long) were aligned to the rn4 genome by BIOGEM (Genomics Data Analysis Services, UCSD) according to the standard Illumina pipeline. The data was analyzed using the HOMER v3.13 software package^80^. After the clonal reads were removed, peaks were defined and annotated and measures for quality control were calculated using default settings designed for ChIP-seq analysis. The UCSC genome browser was used to generate screenshots of Hnf4a binding at specific genes of interest and the Hnf4a peaks were quantified. Peaks were assigned to genes if their transcription start site was the nearest annotated transcription start site as opposed to any other protein-coding gene and the total number of peaks per gene were then quantified and significance was calculated with a two-tailed t test.

### ADME genes and creation of “remote sensing and signaling network”

Genes identified as ADME “core,” “extended,” and “related” (www.pharmaadme.org) were integrated into the transporter gut-liver-kidney network. The top 100 most genes with the most connections, either within each organ, or between organ pairs were identified, transcriptome-wide (extending the lists of ‘single tissue’ and ‘paired tissue’ connections, as above). Of these highly connected genes, 3 additional ADME core genes, 21 additional ADME extended genes, and 6 ADME related genes were identified and were added to the transporter gut-liver-kidney network. To create the ADME gene-focused “remote sensing and signaling” network, we included all ADME genes described above in the network, and their first neighbors (Table S7). This added many oligo-specific and monospecific transporters and DMEs.

## Supplementary information

Supplemental Tables and Figures
