# Supplementary material for: A Network of SLC and ABC Transporter and DME Genes Involved in Remote Sensing and Signaling in the Gut-Liver-Kidney Axis
Source: Sci Rep. 2019 Aug 15;9:11879. doi: 10.1038/s41598-019-47798-x (PMC6695406; doi:10.1038/s41598-019-47798-x)
Supplement: Supplementary file 1 — Supplemental Tables and Figures [file 41598_2019_47798_MOESM1_ESM.pdf]

A Network of SLC and ABC Transporter and DME Genes Involved in Remote Sensing and Signaling in the Gut-Liver-Kidney Axis

Sara Brin Rosenthal, Kevin T. Bush, Sanjay K. Nigam

Supplemental Table S1		
**Families of the 690 SLC and ABC Transporter, as well DME Genes Used for Gut-Liver-Kidney Networl**		
Acyl-CoA synthetase	Guanidinoacetate N-methyltransferase	Solute carrier family 29
Alanine-glyoxylate aminotransferase	Histamine N-methyltransferase	Solute carrier family 3
Alcohol dehydrogenase	Hydroxyacylglutathione hydrolase	Solute carrier family 30
Aldehyde dehydrogenase	Hydroxysteroid dehydrogenase	Solute carrier family 31
Aldehyde oxidase	Hydroxysteroid dehydrogenase like	Solute carrier family 32
Aldo-keto reductase	Indolethylamine N-methyltransferase	Solute carrier family 33
Aralkylamine N-acetyltransferase	Mannosyltransferase	Solute carrier family 34
Arsenite methyltransferase	Methylenetetrahydrofolate reductase	Solute carrier family 35
Arylsulfatase	Monoamine oxidase	Solute carrier family 36
ATP-binding cassette family A	N-acetylglucosaminyltransferase	Solute carrier family 37
ATP-binding cassette family B	N-acetyltransferase	Solute carrier family 38
ATP-binding cassette family C	NAD(P)H quinone dehydrogenase	Solute carrier family 39
ATP-binding cassette family D	N-alpha-acetyltransferase	Solute carrier family 4
ATP-binding cassette family E	Nicotinamide N-methyltransferase	Solute carrier family 41
ATP-binding cassette family F	Paraoxonase	Solute carrier family 43
ATP-binding cassette family G	Phenylethanolamine N-methyltransferase	Solute carrier family 44
Carbohydrate sulfotransferase	Phosphodiesterase	Solute carrier family 45
Carbonyl reductase	Polypeptide N-acetylgalactosaminyltransferase	Solute carrier family 46
carboxylesterase	Rh associated glycoprotein	Solute carrier family 47
Cysteine conjugate-beta lyase	Glucosaminyl (N-acetyl) transferase	Solute carrier family 48
Cysteine rich angiogenic inducer	Glucuronidase	Solute carrier family 49
Cystic fibrosis transmembrane conductance regulator	Glutathione peroxidase	Solute carrier family 5
Cytochrome b5	Glutathione S-transferase	Solute carrier family 50
Cytochrome b561	Glutathione-disulfide reductase	Solute carrier family 51
Cytochrome P450 family 11	Glycine N-choloyltransferase	Solute carrier family 52
Cytochrome P450 family 17	Glycine N-methyltransferase	Solute carrier family 6
Cytochrome P450 family 19	Glycine-N-acyltransferase	Solute carrier family 7
Cytochrome P450 family 2	Granzyme B	Solute carrier family 8
Cytochrome P450 family 20	Solute carrier family 1	Solute carrier family 9
Cytochrome P450 family 21	Solute carrier family 10	Solute carrier organic anion transporter family 1
Cytochrome P450 family 26	Solute carrier family 11	Solute carrier organic anion transporter family 2
Cytochrome P450 family 27	Solute carrier family 12	Solute carrier organic anion transporter family 3
Cytochrome P450 family 39	Solute carrier family 13	Solute carrier organic anion transporter family 4
Cytochrome P450 family 4	Solute carrier family 14	Solute carrier organic anion transporter family 5
Cytochrome P450 family 46	Solute carrier family 15	Solute carrier organic anion transporter family 6

Cytochrome P450 family 7	Solute carrier family 16	Spermidine/spermine N1-acetyltransferase
Cytochrome P450 family 8	Solute carrier family 17	Sulfatase
Dehydrogenase/reductase	Solute carrier family 18	Sulfotransferase family 1
Dihydropyrimidase	Solute carrier family 19	Sulfotransferase family 2
Dihydropyrimidase like	Solute carrier family 2	Sulfotransferase family 4
Dihydropyrimidine dehydrogenase	Solute carrier family 20	Sulfotransferase family 6
Dipeptidase	Solute carrier family 22	Thiopurine S-methyltransferase
Disrupted in renal carcinoma 1	Solute carrier family 23	Thiosulfate sulfurtransferase
Dolichyl-diphosphooligosaccharide-protein glycosyltransferase	Solute carrier family 24	Ubiquitin C-terminal hydrolase like
Epoxide hydrolase	Solute carrier family 25	UDP glucuronosyltransferase family 2
Fatty acid amide hydrolase	Solute carrier family 26	UDP glucuronosyltransferase family 3
Feline leukemia virus subgroup C cellular receptor	Solute carrier family 27	Uncoupling protein
Flavin containing dimethylaniline monoxygenase	Solute carrier family 28	Xanthine dehydrogenase

**Individual 690 DME, SLC and ABC Genes Used For Creation of Gut-Liver-Kidney Network**					
ABCB2	AGXT	SLC7A9	ALG14	SLC39A4	SLC2A7
ABCB3	CYP17A1	SLC7A6	SLC27A3	SULT6B1	SLC2A6
SLC6A2	AS3MT	SLC7A7	SLC27A2	RHBG	ALDH1L1
FLVCR1	CYP1B1	SLC7A4	ABCD2	ABCD4	SLC2A4
SLC27A4	SLC9B2	SLC7A5	NAA38	CHST4	SLC2A3
SLC50A1	CYB5D1	SLC7A2	SLC17A4	SLC6A19	SLC2A2
SLC5A12	CYB5D2	SLC7A3	NAA35	SLC6A18	SLC25A48
SLC27A6	MGAT5B	SLC7A1	SLC17A6	SLC7A6OS	SLC22A31
SLC28A2	SLC35C2	SLC25A35	SLC17A7	CHST7	CES3
SLC43A3	SLC35C1	SLC25A34	NAA30	SLC8A1	CFTR
SLC28A1	TPMT	SLC25A37	SLC17A1	SLC6A12	SLC51A
SLC26A3	SLCO1A2	SLC25A36	SLC17A2	SLC8A3	SLC51B
SLC9C2	NAT14	SLC25A31	SLC17A3	SLC8A2	SULT1B1
ABCD3	NAT10	SLC25A30	ABCA6	SLC6A17	GAMT
SLC9C1	SLC35F3	SLC25A33	ABCA7	SLC6A16	SLC32A1
SLC35E2B	SLC22A18	SLC25A32	ABCA4	SLC6A15	CYB5R4
SLC38A4	SLC22A16	AANAT	ABCA5	SLC6A14	SLC36A1
SLC38A7	SLC22A17	SLC25A39	ABCA2	GALNTL5	PDE3B
SLC38A6	SLC22A14	DPEP1	ABCA3	NAA16	AKR1B10
SLC38A1	SLC22A15	SLC35A4	ABCA1	GALNTL6	NAA25
SLC38A3	SLC22A12	SLC35A5	CCBL1	ACSS2	UGT3A2
SLC38A2	SLC22A13	SLC5A10	ABCA8	ACSS1	CES5A
SLC45A4	SLC22A10	SLC5A11	CCBL2	ABCA13	ALDH18A1
SLC45A1	SLC22A11	SLC35A1	SLC37A3	ALDH7A1	ABCC11
SLC38A8	SLC23A1	SLC35A2	SLC37A1	UCHL5	CHST11
SLC45A3	SLC23A3	SLC35A3	SLC37A4	UCHL1	UGT3A1
SLC45A2	SLC23A2	SLC52A1	DPYD	SLC43A2	SLC16A8
SULT1E1	SLC4A11	SLC14A2	ABCG8	SLC43A1	MTHFR
SLC17A8	SLC4A10	SLC14A1	SLC22A18AS	CYP1A2	SLC13A1
CYP26A1	SLC5A1	CES4A	SULF2	GALNT12	SLC13A2
SLC17A5	SLC5A2	SLC35E4	SULF1	GALNT13	SLC13A3
HSD17B12	SLC5A3	SLC52A3	DPYS	GALNT10	SLC13A4
HSD17B13	SLC5A4	SLC26A1	SLCO5A1	GALNT11	SLC6A20

HSD17B10	SLC5A5	SLC26A2	HSD17B1	GSTM1	SLC6A13
HSD17B11	SLC5A6	ABCB10	HSD17B2	GSTM2	SLC6A11
HSD17B14	SLC5A8	SLC26A4	HSD17B3	NAA11	CYP7A1
ALDH3A2	SLC5A9	SLC26A6	HSD17B4	GSTM4	ADH7
GSTZ1	NAA40	SLC26A7	HSD17B6	GSTM5	SLC8B1
ALDH3A1	ABCB1	NQO1	DHRS7B	SLC35D3	ADH5
MGST2	ABCB7	SLC26A9	ALDH3B2	SLC35D2	ADH4
MGST1	ABCB6	SLC25A40	ALDH3B1	SLC35D1	GALNT14
TST	ABCB5	ABCG1	CYR61	SAT1	SLC7A14
CYP2U1	ABCB4	SLC25A42	SLC38A9	SAT2	SLC48A1
CYP11A1	ABCB9	SLC25A43	SLC24A4	CYP19A1	GNMT
CYP27B1	ABCB8	SLC25A44	SLC24A5	SLCO1B1	AKR1D1
HNMT	DIRC2	SLC25A45	SLC24A2	SLCO1B3	SLC33A1
SLC26A8	MFSD7	SLC25A46	SLC24A3	SLCO1B7	DDOST
ALDH8A1	SLC13A5	SLC25A47	SLC24A1	ALDH1A1	SLC18B1
SLC39A9	MGAT1	CHST8	SLC35F5	ALDH1A2	HAGH
CHST1	MGAT2	SLC3A2	XDH	ALDH1A3	SLC9A3R2
SLC39A1	MGAT3	SLC3A1	SLCO4C1	SLC22A4	SLC9A3R1
CHST3	MGAT5	CYP2E1	ALDH16A1	GCNT7	SLC25A38
SLC39A3	CYP11B2	NNMT	SULT1C2	GCNT1	SLC38A10
CHST5	UCP1	SULT2B1	DHRS13	SLC35B1	SLC35E1
SLC39A5	UCP2	SLCO2B1	ALDH2	GCNT2	SLC35E2
SLC39A6	UCP3	GSTM3	SLC39A13	CBR4	SLC35E3
SLC39A7	NAT8L	SLC35F4	DHRS11	CBR1	HSD11B2
GSTO2	SLC1A6	SLC35F6	SLC2A1	SLC35B3	HSD11B1
GSTO1	ABCF2	SLC35F1	HSD3B7	CBR3	SLC2A5
SULT4A1	SLC16A9	GCNT3	HSD3B1	GPX2	ALDH6A1
GZMB	CHST10	ARSB	HSD3B2	SLC16A11	GGT1
ABCB11	CHST13	SLC35F2	SLC9B1	SLC16A10	GGT6
GPX1	CHST12	BAAT	ALDH5A1	SLC16A13	GGT7
SLC22A5	CHST15	CYB5B	ABCE1	SLC16A12	ALDH1B1
SLC22A6	CHST14	ARSK	ALG8	FAAH	ABCC12
SLC35B2	SLC16A2	SLC25A21	ALG9	SLC22A1	ABCC10
GPX5	SLC16A5	MGAT4A	CYB5R3	SLC44A1	SLCO1C1
SLC35B4	SLC16A4	PNMT	CYB5R2	SLC44A2	RHCG
SLC22A2	SLC16A7	MGAT4C	CYB5R1	SLC44A3	SLC7A10
GPX6	SLC16A6	MGAT4B	DHRS7C	SLC44A4	SLC7A11
SLC18A3	GSTP1	SLC1A4	ALG2	GPX7	SLC7A13
GPX8	SLC6A3	CYB561D1	ALG3	SLC22A3	GLYAT
SLC18A1	FLVCR2	CYP11B1	ALG1	SLCO2A1	SLC4A1AP
ABCA9	SLC6A1	SLC1A7	ALG6	SLC20A2	GSTK1
SLC22A8	SLC6A7	SLC1A1	ALG5	NAA50	SLC25A4
SLC22A9	SLC6A6	SLC1A2	SLC29A4	PON1	SLC25A22
CYP46A1	SLC6A5	SLC1A3	SLC29A3	PON3	SLC25A23
AOX1	SLC6A4	GSTA4	SLC29A2	PON2	SLC25A20
SLC2A14	SLC6A9	NQO2	SLC44A5	SLC2A4RG	SLCO6A1
SLC2A13	SLC30A9	GSTA2	SLC11A2	GSTCD	SLC25A26
SLC2A12	SLC30A5	GSTA3	SLC11A1	ABCC8	SLC25A27
SLC2A11	SLC30A4	SLC25A19	CYB561	ABCC9	SLC25A24
SLC2A10	SLC30A7	SLC25A18	CYP26B1	ABCC4	SLC25A25
SLC4A5	SLC30A6	SLC25A17	ABCG2	ABCC5	SLC15A2
SLC4A4	SLC30A1	SLC25A16	SLC46A2	ABCC6	SLC15A3
SLC4A7	SLC30A3	SLC25A15	SLC46A3	ABCC1	SLC25A28
SLC4A1	SLC30A2	SLC25A14	SLC46A1	ABCC2	SLC25A29
SLC4A3	SLC35G2	SLC25A13	DPYSL4	ABCC3	SLC15A4
SLC4A2	SLC35G3	SLC25A12	DPYSL5	GSR	SLC15A5
SLC4A9	SLC35G1	SLC25A11	DPYSL2	SLC52A2	RHAG
SLC4A8	SLC35G6	SLC25A10	DPYSL3	SLC27A1	GPX4
SLC25A53	SLC35G5	SLC15A1	NAA20	SLC39A12	SLC1A5

SLC25A52	ACSM1	DHRS7	GALNT9	CYB561D2	SLC31A1
SLC25A51	GPX3	DHRS4	GALNT4	SLC39A10	ACSBG1
GALNT7	ACSM3	DHRS2	GALNT6	SLC39A11	ACSBG2
SLC22A23	ACSM5	DHRS3	CYP2R1	SLC39A14	SLC9A4
SLC30A10	GUSB	DHRS1	GALNT1	SLC34A1	SLC9A5
SLC36A4	FMO2	SLC12A5	GALNT2	SLC34A3	SLC9A6
SLC36A3	FMO3	SLC12A4	GALNT3	SLC34A2	SLC9A7
SLC36A2	FMO1	SLC12A7	CYP8B1	CYP39A1	ABCF3
SLC22A25	FMO4	SLC12A6	COMTD1	SLC26A10	SLC9A1
SLC22A24	FMO5	SLC12A1	CYP27A1	SLC26A11	SLC9A2
NAT8	SLC25A3	SLC12A3	MAOB	SLCO3A1	SLC9A3
SLC25A41	SLC25A2	SLC12A2	MAOA	AGXT2	SLC9A8
NAT2	SLC25A1	SLC47A2	ABCG4	HSDL1	SLC9A9
NAT1	SLC25A6	SLC47A1	CYB5RL	HSDL2	ALG10B
SULT1A1	SLC25A5	SLC12A9	EPHX1	ACSL3	CYP2S1
NAT6	ACSF2	SLC12A8	EPHX2	ACSL1	SLC10A3
CYP20A1	ACSF3	DHRSX	EPHX3	ACSL6	SLC10A2
ABCD1	ABCA10	SLC41A1	EPHX4	ACSL4	SLC10A1
ALDH9A1	SLC39A8	SLC41A3	INMT	ACSL5	CHST2
SLC27A5	ABCA12	SLC41A2	UGT2A3	ALDH1L2	SLC10A7
ALG11	NAA15	SLC22A7	CYP4B1	SLC19A2	SLC10A6
ALG12	ALDH4A1	SLC2A9	ABCG5	SLC19A3	SLC10A5
ALG13	NAA10	SLC2A8	SLC19A1	SLC7A8	SLC10A4

Table S1. SLC and ABC Transporters, as well as DME Genes and Gene Families Used to Create Gut-Liver-Kidney Network. A co-expression analysis was performed in which 20,000 genes were correlated across multiple human tissues (20k x 20k). This 20,000 gene co-expression network was then filtered for Phase I and Phase II drug metabolizing enzymes (DMEs), as well as SLC and ABC transporters comprising multiple gene families resulting in a list of 690 SLC, ABC and DME genes which are co-expressed across multiple organs. This list of SLC and ABC transporters, as well as the DMEs (and their families) was compiled from lists derived from a number of human and rodent studies or databases and are listed in Table S1.

	Tissue Pairs		
Gene	('kidney', 'liver')	('liver', 'gut')	('kidney', 'gut')
DAO	51	0	0
DMGDH	51	0	0
HAO2	48	0	0
CLYBL	47	4	4
SLC27A2	46	1	1
GLYATL1	46	0	0
BHMT	45	0	0
ECI2	45	0	0
ACAT1	42	0	0
AFP	42	0	0
EHHADH	42	0	0
ASS1	41	1	1
SHMT1	40	0	0
ACSM2A	39	0	0
ACSM2B	38	0	0
ALDOB	37	15	15
AIG1	37	1	1
BHMT2	35	0	0
ENSG00000226215	35	0	0
ENSG00000231974	35	0	0

	Tissue Pairs		
Gene	('kidney', 'gut')	('liver', 'gut')	('kidney', 'liver')
ACY3	31	2	2
GLOD5	27	4	4
XPNPEP2	26	1	1
SLC6A19	24	1	1
TTC38	23	27	23
ENPEP	23	1	1
KCNK5	21	0	0
SLC28A1	18	18	21
HNF1A	18	5	5
SLC7A9	18	1	1
KHK	17	17	32
DHDH	16	1	1
DPEP1	16	0	0
ALDOB	15	15	37
GIPC2	15	1	1
MME	15	1	1
GK	14	5	5
LRRC19	14	1	1
TMEM139	14	0	0
PBLD	13	13	22

	Tissue Pairs		
Gene	('liver', 'gut')	('kidney', 'gut')	('kidney', 'liver')
NR1I2	35	1	1
PCK2	32	11	11
SEC16B	29	1	1
SLC46A3	29	1	1
TTC38	27	23	23
ABCG8	26	1	1
CYP4F2	22	5	5
RP11-407N17.3	22	9	9
IGSF23	21	1	1
HNF4A	20	5	5
SULT1E1	20	1	1
NR5A2	19	0	0
CYP3A5	18	1	1
SLC28A1	18	18	21
KHK	17	17	32
MTTP	17	1	1
CYP2C18	16	1	1
PLA2G12B	16	7	7
ALDOB	15	15	37
FABP1	15	0	0

Table S2: Genes with the most cross-tissue connections, between all pairs of tissues in the gut-liver-kidney subnetwork. The top 20 genes are displayed, ranked by number of connections to genes in the (A) kidney-liver pair, (B) kidney-gut pair, and (C) liver-gut pair.

A

	liver	kidney	gut
F11	74	8	6
HAAO	74	5	6
ACADSB	73	11	2
ACSM5	73	9	3
CPN2	73	6	3
MUT	73	11	3
OIT3	73	3	6
PLG	73	8	4
ADH6	72	7	6
AGMO	72	5	6
APOC3	72	1	6
CFI	72	10	1
F12	72	1	3
FCN2	72	1	3
GJB1	72	7	7
MTHFD1	72	8	0
NR1I3	72	4	5
PIPOX	72	17	1
SLC22A7	72	8	2
TMEM176B	72	8	7

B

	liver	kidney	gut
HOGA1	9	57	1
OGDHL	7	57	2
L2HGDH	2	57	0
RNF152	2	57	1
SFXN2	2	57	2
BHMT2	35	56	1
ACMSD	20	56	1
BPHL	11	56	7
CRYL1	8	56	10
AGMAT	7	56	7
MSRA	7	56	5
PDZK1	5	56	6
NOX4	1	56	4
SLC13A3	1	56	2
SLC22A8	1	56	4
TMEM52B	1	56	0
C9orf66	0	56	3
KLHDC7A	0	56	0
SOST	0	56	1
GALM	4	55	9

C

	liver	kidney	gut
TM6SF2	3	1	52
PRAP1	5	1	50
ANPEP	0	1	50
HNF4G	0	1	50
BAIAP2L2	0	0	50
DGAT1	0	0	49
TMEM150B	0	0	49
SLC5A9	0	1	48
BTNL3	0	0	48
CDHR2	0	1	47
ENSG000002	0	1	47
MS4A10	0	1	47
APOBEC1	0	0	47
BTNL8	0	0	47
FAM132A	0	0	47
MYO1A	0	0	47
SI	0	0	47
CBR1	4	1	46
HTR1D	0	0	46
MEP1B	0	0	46

Table S3: Genes which are the most tissue-specific in the gut-liver-kidney subnetwork. Top 20 genes displayed, sorted by A) number of connections to liver SLC-DME-ABC genes, B) number of connections to kidney SLC-DME-ABC genes, and C) number of connections to gut SLC-DME-ABC genes.

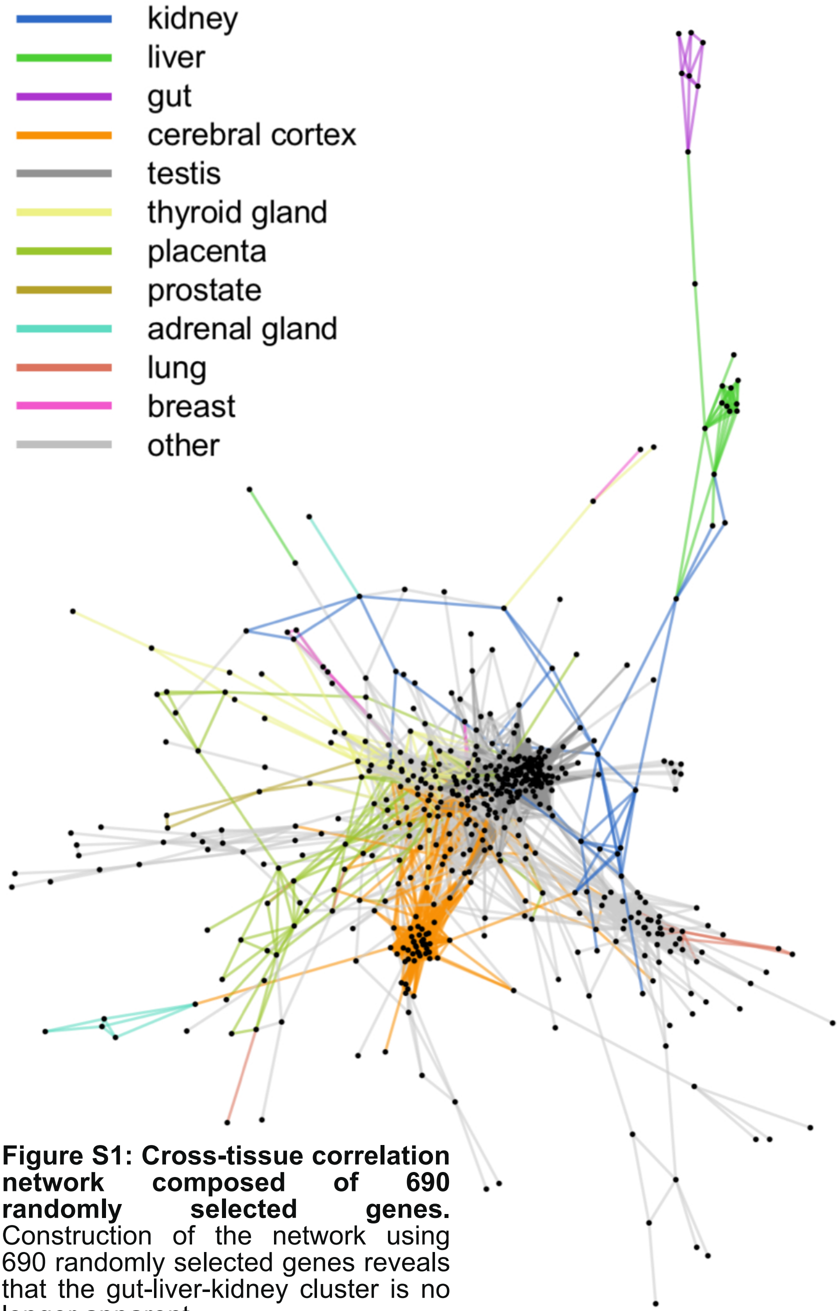

Figure S1: Cross-tissue correlation network composed of 690 randomly selected genes. Construction of the network using 690 randomly selected genes reveals that the gut-liver-kidney cluster is no longer apparent.

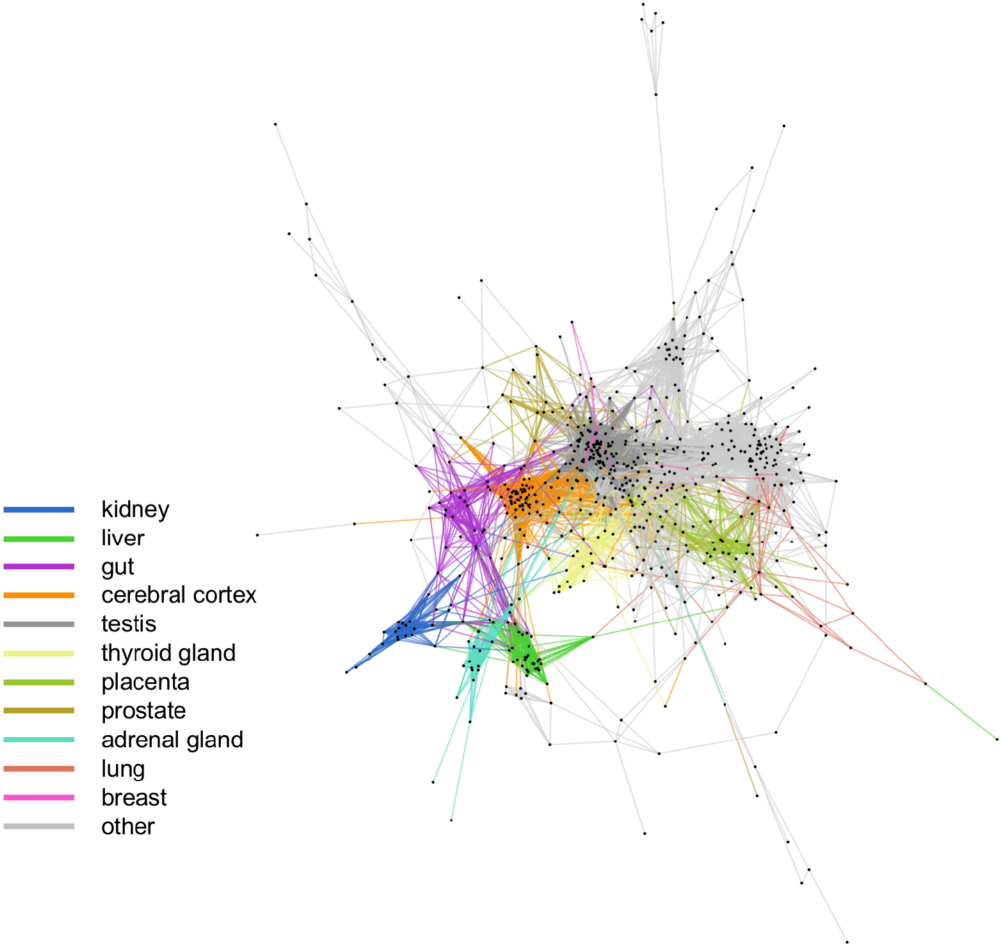

kidney
liver
gut
cerebral cortex
testis
thyroid gland
placenta
prostate
adrenal gland
lung
breast
other

Figure S2: Cross-tissue correlation network composed of 690 GPCR type a genes (non-olfactory). Construction of the network using 690 GPCRs demonstrates that tut-liver-kidney cluster is much less apparent than if the network is constructed using the 690 SLC-DME-ABC genes.

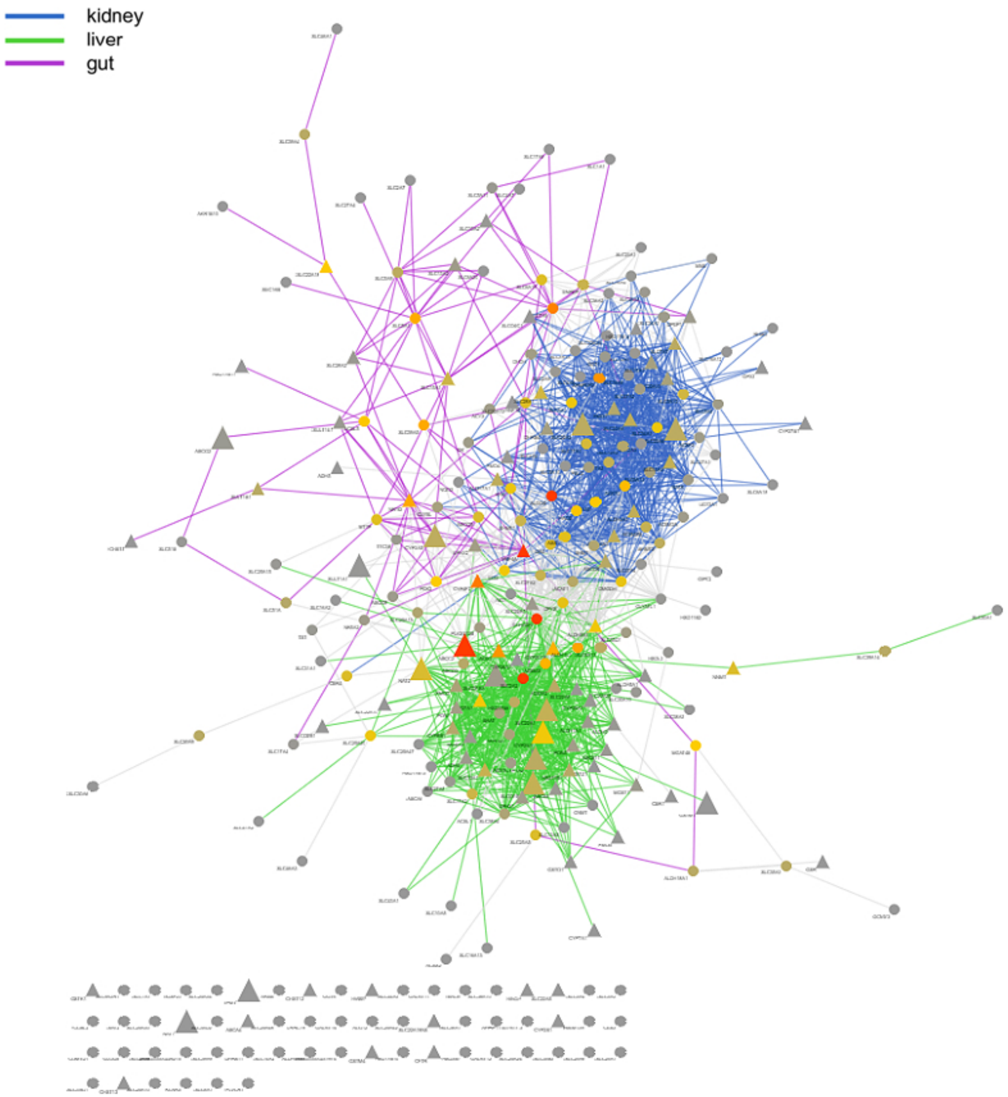

kidney
liver
gut

Figure S3: Filtering of gut-liver-kidney network in a robust protein-protein interactome reveals biological relevance of the network. The gut-liver-kidney co-expression network was filtered by protein-protein interactions in order to prioritize edges with likely biological relevance. In this case, an edge is only present if the two genes connected by the edge are 1) coexpressed in human protein atlas database and 2) they are also have been found to functionally interact. In the final filtered network, there were 1959 edges reduced from more than 6000 in the unfiltered network. The filtered network retains the tissue-specific clustering which was evident in the co-expression network

TABLE S4<br>HNF4α					
Gene	Correlation Coefficient	Expression Down in *Hnf4α*KO			ChIP-Seq (at least 1 peak ≥50)
		Liver	Colon	Small Intestine	
HSD17B11	0.657	✓	✓	✓	✓
ALDH2	0.453	✓	✓		✓
SLC39A14	0.517	✓	✓		✓
SLC25A15	0.494	✓	✓		✓
ABCD3	0.631	✓			✓
ACSM5	0.438	✓			✓
ALDH8A1	0.537	✓			✓
DHRS4	0.554	✓			✓
DPYS	0.512	✓			✓
EPHX2	0.656	✓			✓
SLC22A18	0.600	✓			✓
SLC27A2	0.506	✓			✓
SLC2A2	0.487	✓			✓
SLC31A1	0.514	✓			✓
SLC35D1	0.697	✓			✓
SLC9A3R1	0.599	✓			✓
CBR1	0.681	✓			✓
SLC25A1	0.517	✓			✓
SLC30A10	0.666	✓			✓
SLC37A4	0.618	✓			✓
SULT1A1	0.613	✓			✓
TST	0.829	✓			✓
MGST2	0.556	✓		✓	
GGT1	0.436	✓		✓	
SLC25A20	0.689	✓		✓	
SLC23A1	0.441	✓		✓	
HSD3B7	0.454	✓	✓		
SLC39A4	0.622	✓	✓		
SLC39A5	0.905	✓	✓		
ACSL5	0.648	✓			
GSTK1	0.575	✓			
ABCC2	0.618	✓			
ABCG5	0.594	✓			
CYP27A1	0.484	✓			
SLC25A13	0.494	✓			
CES3	0.815	✓			
SLC6A19	0.642		✓		✓
SLC13A2	0.651		✓		✓
SLC7A9	0.582		✓		✓
DHRS11	0.688		✓		✓
GSR	0.463		✓		✓
XDH	0.488		✓		✓
CYP2S1	0.430		✓		
DPEP1	0.509		✓		
FMO5	0.547		✓		
GGT6	0.651		✓		
SLC23A3	0.461		✓		
GSTA2	0.535		✓		
MGAT4B	0.737		✓		
SLC35D2	0.689		✓		
ABCC3	0.443			✓	✓
SLC1A1	0.482			✓	✓
MAOA	0.595			✓	
ALDH18A1	0.444				✓
ABCG2	0.453				✓
ABCC6	0.641	✓			✓
ABCG8	0.629				✓
ACSS2	0.651	✓			✓
SLC16A13	0.522				✓
SLC25A10	0.597	✓			✓
SLC25A34	0.517				✓

SLC25A5	0.716				✓
SLC27A1	0.450				✓
SLC5A11	0.459				✓
SLC5A9	0.546				✓
SULT1B1	0.732	✓			✓
SLC15A1	0.498				✓
SLC22A7	0.503	✓			✓
SLC28A1	0.709				✓
SLC34A3	0.440				✓
SLC41A2	0.629				✓
SLC44A3	0.584				✓
SLC5A1	0.514				✓
SLC6A20	0.437				✓
AKR1B10	0.469				
ALDH1A1	0.444	✓			
GALNT12	0.438				
GCNT3	0.562				
NAA25	0.488				
NAT10	0.461				
SLC12A4	0.434				
SLC26A3	0.506	✓			
SLC35A3	0.567	✓			
SLC35G1	0.494				
SLC46A3	0.647	✓			
SLC6A12	0.434				
SLC7A6OS	0.424	✓			
UGT2A3	0.759	✓			
UGT3A1	0.489	✓			
ALDH1L1	0.473	✓			
CHST5	0.657				
COMTD1	0.505				
GSTM4	0.532				
HNF1A	0.857	✓			
HSD11B2	0.641				
NAT1	0.552				
NAT2	0.633	✓			
SAT2	0.491	✓			
SLC10A5	0.726				
SLC22A18AS	0.721				
SLC28A2	0.564				
SLC2A7	0.436				
SLC51B	0.622				
SLC5A4	0.427				
SULT1E1	0.633				

Table S4: Wet-lab validation of genes highly associated with HNF4a. The expression of 108 genes, which were found to be highly associated with HNF4α in our analysis, was examined in available microarray of HNF4α-tissue-specific knockouts analyses or in ChIP-Seq examination of adult kidney. In knockout data, genes whose expression was down-regulated in the absence of HNF4α are indicated by a checkmark. In the ChIP-Seq analysis of HNF4α in adult kidney, genes displaying at least one peak greater than 50 are indicated by a checkmark.

TABLE S5<br>HNF1α			
Gene	Correlation Coefficient	Expression Down in *Hnf1α*KO	
		Liver (≥3-fold)	Intestine
ABCC6	0.621	✓	✓
ABCD3	0.483	✓	✓
ABCG5	0.551	✓	✓
ABCG8	0.559	✓	✓
AKR1B10	0.497	✓	
ALDH6A1	0.441	✓	
ALDH8A1	0.457	✓	
CBR1	0.748	✓	
CHST5	0.502	✓	
DHRS11	0.635	✓	
DHRS4	0.722	✓	
DPEP1	0.775	✓	
EPHX2	0.610	✓	
GGT1	0.648	✓	
GSTA2	0.480	✓	
HNF4A	0.857	✓	
HSD17B11	0.524	✓	
HSD3B7	0.497	✓	
NAT8	0.483	✓	
SLC10A2	0.554	✓	
SLC13A2	0.808	✓	
SLC13A3	0.460	✓	
SLC15A1	0.595	✓	
SLC16A13	0.510	✓	
SLC1A1	0.558	✓	
SLC22A12	0.562	✓	
SLC22A13	0.534	✓	
SLC22A5	0.430	✓	
SLC22A6	0.576	✓	
SLC22A8	0.502	✓	
SLC23A1	0.618	✓	
SLC23A3	0.629	✓	
SLC25A10	0.701	✓	
SLC25A34	0.618	✓	
SLC27A2	0.442	✓	
SLC31A1	0.426	✓	
SLC34A1	0.563	✓	
SLC34A3	0.699	✓	
SLC35D2	0.556	✓	
SLC37A4	0.604	✓	
SLC39A4	0.720	✓	
SLC39A5	0.940	✓	
SLC3A1	0.486	✓	
SLC44A3	0.485	✓	
SLC46A3	0.571	✓	
SLC51A	0.535	✓	
SLC51B	0.617	✓	
SLC5A10	0.557	✓	
SLC5A11	0.609	✓	
SLC5A12	0.466	✓	
SLC5A9	0.673	✓	
SLC6A18	0.542	✓	
SLC7A7	0.527	✓	
SLC7A9	0.775	✓	
SULT1A1	0.488	✓	
SULT1B1	0.526	✓	
SULT1E1	0.639	✓	
UGT2A3	0.749	✓	
UGT3A1	0.495	✓	
ABCC2	0.538		✓
ABCG2	0.457		✓
ACSF2	0.504		✓
ACSL5	0.578		
ACSS2	0.450		
AGXT2	0.445		
ALDH1L1	0.429		
ALDH4A1	0.559		
CES3	0.564		

COMTD1	0.542		
DPYS	0.466		
GGT6	0.579		
GSTK1	0.546		
GSTM4	0.577		
HSD11B2	0.563		
MAOA	0.536		
MGAT4B	0.604		
NQO2	0.475		
SAT2	0.635		
SLC10A5	0.497		
SLC17A4	0.555		
SLC17A8	0.436		
SLC22A18	0.642		
SLC22A18AS	0.546		
SLC25A1	0.587		
SLC25A20	0.506		
SLC25A5	0.637		
SLC27A4	0.432		
SLC28A1	0.797		
SLC28A2	0.607		
SLC2A7	0.494		
SLC30A10	0.484		
SLC35G1	0.508		
SLC47A2	0.495		
SLC5A1	0.590		
SLC5A2	0.526		
SLC5A4	0.523		
SLC6A13	0.482		
SLC6A19	0.845		
SLC6A20	0.554		
SLC9A3R1	0.724		
TST	0.653		

Table S5: Wet-lab validation of genes highly associated with HNF1a. The expression of 101 genes, which were found to be highly associated with HNF1α in our analysis, was examined in available microarray of HNF1α knockouts analyses. In knockout data, genes whose expression was down-regulated in the absence of HNF1α are indicated by a checkmark.

TABLE S6<br>PXR			
**Gene**	**Correlation Coefficient**	**Expression Down In Liver of *Pxr*KO**	**Expression Up In Liver Following Treatment with PXR activator**
ABCB11	0.472	✓	
ABCC2	0.715	✓	
ABCC3	0.438	✓	✓
ABCC6	0.509	✓	
ABCD3	0.527	✓	
ABCG5	0.709	✓	
ABCG8	0.753	✓	
ACSL5	0.756	✓	
ACSM5	0.468	✓	
ACSS2	0.541	✓	
ADH4	0.560	✓	
AGXT	0.493	✓	
AKR1B10	0.511	✓	
AKR1D1	0.527	✓	✓
ALDH18A1	0.465	✓	
BAAT	0.522	✓	
CYP1A2	0.442	✓	
CYP27A1	0.573	✓	
CYP2E1	0.511	✓	
CYP8B1	0.537	✓	
DHRS1	0.441	✓	
EPHX2	0.571	✓	
GCNT3	0.540	✓	
GNMT	0.438	✓	
GSTK1	0.533	✓	
GSTZ1	0.457	✓	
HNF4A	0.815	✓	
HNF4G	0.749	✓	
HSD11B1	0.485	✓	
HSD17B11	0.742	✓	
HSD17B6	0.490	✓	✓
HSD3B7	0.446	✓	
MAOA	0.536	✓	
MGST1	0.443	✓	
NR1H4	0.724	✓	
NR1I3	0.551	✓	
NR5A2	0.883	✓	
PON1	0.529	✓	
SLC10A1	0.542	✓	
SLC13A2	0.571	✓	✓
SLC16A13	0.464	✓	
SLC17A2	0.510	✓	
SLC22A18	0.567	✓	
SLC25A13	0.512	✓	
SLC25A20	0.774	✓	
SLC25A34	0.543	✓	
SLC25A5	0.527	✓	
SLC27A5	0.503	✓	
SLC2A2	0.596	✓	
SLC30A1	0.487	✓	
SLC31A1	0.535	✓	
SLC35A3	0.483	✓	
SLC35D1	0.719	✓	
SLC35G1	0.559	✓	
SLC38A3	0.489	✓	
SLC38A4	0.537	✓	
SLC39A14	0.619	✓	
SLC39A4	0.585	✓	
SLC39A5	0.758	✓	
SLC46A3	0.816	✓	
SLC51A	0.747	✓	
SLC5A9	0.671	✓	
SLC6A19	0.491	✓	
SLC7A9	0.494	✓	
SLC9A3R1	0.483	✓	
SULT1B1	0.728	✓	
UGT2A3	0.663	✓	

ALDH1A1	0.500		✓
GSTA2	0.435		✓
GSTM4	0.529		✓
ABCB4	0.468		
ABCG2	0.575		
CBR1	0.695		
CES3	0.685		
CHST5	0.633		
DHRS11	0.727		
FMO3	0.521		
FMO5	0.650		
GSTO1	0.487		
HNF1A	0.664		
HSD17B13	0.520		
MGAT4B	0.735		
MGST2	0.643		
NAT1	0.452		
NAT2	0.798		
PON3	0.520		
SLC10A2	0.432		
SLC10A5	0.755		
SLC13A5	0.514		
SLC15A1	0.655		
SLC17A4	0.865		
SLC17A8	0.477		
SLC22A1	0.524		
SLC22A10	0.461		
SLC22A18AS	0.512		
SLC22A25	0.485		
SLC22A7	0.442		
SLC22A9	0.527		
SLC25A1	0.436		
SLC25A15	0.657		
SLC25A47	0.437		
SLC28A1	0.547		
SLC28A2	0.663		
SLC2A7	0.512		
SLC30A10	0.768		
SLC35D2	0.636		
SLC37A4	0.479		
SLC41A2	0.722		
SLC51B	0.618		
SLC5A1	0.607		
SLC5A4	0.525		
SLC6A20	0.551		
SLCO1B1	0.510		
SLCO1B3	0.467		
SULT1A1	0.698		
SULT1E1	0.801		
TST	0.711		
XDH	0.575		

Table S6: Wet-lab validation of genes highly associated with PXR. The expression of 118 genes, which were found to be highly associated with PXR in our analysis, was examined in available microarray of either PXR knockouts or in animals treated with the PXR agonist PCN. In knockout data, genes whose expression was down-regulated in the absence of PXR are indicated by a checkmark, while in the PCN-treated data, genes displaying increased expression in the presence of the agonist are indicated by a checkmark.

Supplemental Table S7				
	PharmaADME Designation			First Neighbor
Gene	Core	Extended	Related	
CYP2C9	X			
CYP2E1	X			
SLC22A1	X			
CYP1A2	X			
SLCO1B1	X			
SLC22A2	X			
SLC22A6	X			
NAT2	X			
SLCO1B3	X			
UGT2B7	X			
CYP3A5	X			
UGT1A1	X			
SULT1A1	X			
ABCC2	X			
ABCG2	X			
CAT		X		
NR1I3		X		
MAT1A		X		
SERPINA7		X		
SOD1		X		
NR1I2		X		
HNF4A		X		
PPARA		X		
CYP4A11		X		
ADH4		X		
ADH6		X		
UGT2B4		X		
FMO3		X		
HSD11B1		X		
SLC22A8		X		
ADH1A		X		
ALDH8A1		X		
CYP8B1		X		
PON1		X		
SLC10A1		X		
ALDH6A1		X		
CYP27A1		X		
SLC13A3		X		

FMO1		X		
ALDH4A1		X		
PON3		X		
AOX1		X		
CYP4F2		X		
SLC13A1		X		
CYP2C18		X		
SLC22A13		X		
GSTA1		X		
GSTA2		X		
ALDH1A1		X		
SLC22A7		X		
SLC7A7		X		
EPHX1		X		
ABCB11		X		
CYP4F11		X		
CYP39A1		X		
SLC22A9		X		
ALDH7A1		X		
ABCC6		X		
UGT1A4		X		
ABCB4		X		
DHRS4		X		
ALDH2		X		
FMO4		X		
CES2		X		
SLC28A1		X		
SLC15A1		X		
UGT2B10		X		
SLC22A12		X		
UGT1A9		X		
CYP26A1		X		
ALDH5A1		X		
MGST1		X		
SULT1A2		X		
UGT1A6		X		
SLCO4C1		X		
SULT1E1		X		
GSTO1		X		
EPHX2		X		
GSTZ1		X		

FMO5		X		
SLC22A10		X		
GPX3		X		
CYP2J2		X		
CBR1		X		
SLC13A2		X		
CYP4F12		X		
HSD17B14		X		
SLC10A2		X		
SULT1B1		X		
DPEP1		X		
MGST2		X		
SLC28A2		X		
SLC22A18		X		
CYP27B1		X		
CHST5		X		
NNMT		X		
DHRS4L2		X		
HSD17B11		X		
CYP7A1		X		
HAGH		X		
APOA2			X	
TTR			X	
PLG			X	
BDH2			X	
CRYZ			X	
UROC1			X	
SLC2A2				X
EHHADH				X
ACSM5				X
HSD17B6				X
GLYAT				X
HAO2				X
ALDH1L1				X
AGXT2				X
SLC17A1				X
ALDOB				X
SLC17A3				X
AKR1D1				X
DMGDH				X
DAO				X

BAAT				X
AGXT				X
DPYS				X
SLC12A3				X
BHMT				X
SLC3A1				X
SLC34A1				X
SLC6A13				X
SLC27A5				X
SLC38A4				X
ASS1				X
SHMT1				X
SLC5A12				X
NAT8				X
SLC47A2				X
SLC25A47				X
BHMT2				X
SLC5A10				X
ACAT1				X
XPNPEP2				X
SLC5A2				X
ECI2				X
SLC7A13				X
KHK				X
SLC7A9				X
GLYATL1				X
SLC12A1				X
SLC17A2				X
SLC6A12				X
PBLD				X
SLC5A1				X
ACSM2A				X
ACSM2B				X
PCK2				X
SLC22A24				X
PLA2G12B				X
SLC27A2				X
HNF1A				X
HSD17B13				X
SLC2A9				X
DHDH				X

SLC22A25				X
ACSL5				X
SLC4A4				X
SLC4A9				X
ACSF2				X
SLC6A19				X
GNMT				X
SLC5A9				X
SLC16A9				X
SLC47A1				X
SLC13A5				X
UGT3A1				X
SLC39A5				X
ENPEP				X
GGT1				X
SLC25A20				X
SLC25A13				X
ABCG5				X
MTTP				X
SLC36A2				X
ABCA6				X
SLC16A4				X
NQO2				X
ACSL1				X
CLYBL				X
SLC23A3				X
SLC43A1				X
SLC35D1				X
NR5A2				X
ABCG8				X
TTC38				X
UGT2A3				X
MME				X
ACY3				X
MGAT4B				X
HSDL2				X
ABCD3				X
SLC6A20				X
SLC39A14				X
SLC17A8				X
TST				X

SLC16A12				X
SLC25A5				X
HSD17B10				X
SLC31A1				X
CES3				X
SLC51A				X
SLC51B				X
AKR1B10				X
SLC39A4				X

Table S7: Genes comprising tentative ADME gene-based remote sensing and signaling network for gut-liver-kidney axis. List of genes comprising remote sensing and signaling network. ADME genes from pharmaadme.org found within the list are shown and whether they are "core," "extended," or "related" is indicated by an X in the appropriate column. First neighbors are also indicated by an X.

Here we provide a brief overview of the strategy used in creating this final remote sensing and signaling network. Initially, a co-expression analysis was performed in which 20,000 genes were correlated across multiple human tissues (20k x 20k). This 20,000 gene co-expression network was then filtered for Phase I and Phase II drug metabolizing enzymes (DMEs), as well as SLC and ABC transporters resulting in a list of 690 SLC, ABC and DME genes which are co-expressed across multiple organs. This list of SLC and ABC transporters, as well as the DMEs was compiled from lists derived from a number of human and rodent studies or databases. Among the various tissue-specific clusters, the gut-liver-kidney (GLK) cluster was found to comprise one of the highest inter-connected sets. In order to potentially identify genes involved in the regulation/modulation of the network, the GLK subnetwork was expanded to include genes from the entire 20,000 gene co-expression network that displayed the most paired tissue connections to/ within the GLK network. The expanded GLK-subnetwork was then filtered for functional/biological relevance using a robust database of authentic protein-protein interactions. Genes contained within this network were then identified as ADME genes using information contained in the PharmADME database. This resulted in a network of 282 genes which, by taking just the "first neighbors" of the ADME genes, was subsequently reduced to 211 genes. This final list of 211 genes is the "Remote Sensing and Signaling" (RSSH) network.
